# Enhanced-Sampling Simulations for the Estimation of Ligand Binding Kinetics: Current Status and Perspective

**DOI:** 10.3389/fmolb.2022.899805

**Published:** 2022-06-08

**Authors:** Katya Ahmad, Andrea Rizzi, Riccardo Capelli, Davide Mandelli, Wenping Lyu, Paolo Carloni

**Affiliations:** ^1^ Computational Biomedicine (IAS-5/INM-9), Forschungszentrum Jülich, Jülich, Germany; ^2^ Atomistic Simulations, Istituto Italiano di Tecnologia, Genova, Italy; ^3^ Department of Applied Science and Technology (DISAT), Politecnico di Torino, Torino, Italy; ^4^ Warshel Institute for Computational Biology, School of Life and Health Sciences, The Chinese University of Hong Kong (Shenzhen), Shenzhen, China; ^5^ School of Chemistry and Materials Science, University of Science and Technology of China, Hefei, China; ^6^ Molecular Neuroscience and Neuroimaging (INM-11), Forschungszentrum Jülich, Jülich, Germany

**Keywords:** kinetics, drug discovery, QM/MM, parallel computing, machine learning, enhanced sampling, molecular dynamics

## Abstract

The dissociation rate (*k*_off_) associated with ligand unbinding events from proteins is a parameter of fundamental importance in drug design. Here we review recent major advancements in molecular simulation methodologies for the prediction of *k*_off_. Next, we discuss the impact of the potential energy function models on the accuracy of calculated *k*_off_ values. Finally, we provide a perspective from high-performance computing and machine learning which might help improve such predictions.

## 1 Introduction

The kinetics of drugs unbinding from proteins is an important parameter for the drugs’ efficacy. (Pan et al., 2013; Copeland, 2021). Indeed, the drug-target residence time (Copeland, Pompliano and Meek, 2006) defined as the inverse of the dissociation rate *k*_off_, has emerged as an effective surrogate measure of *in vivo* target occupancy, and it has been shown to correlate with clinical efficacy (Guo et al., 2012; Lee et al., 2019; Van Der Velden et al., 2020) along with other factors (e.g., association rates (Folmer, 2018; Lee et al., 2019) and target saturation (de Witte et al., 2018)). Residence time has been related not only to long-lasting pharmacodynamics but also to the reduced toxicity of specific inhibitors (Vauquelin et al., 2012).

Experimental approaches (most often combined with computations) measure ligand affinities and provide ligand binding poses for structure-based drug design campaigns (Durrant and McCammon, 2011; De Vivo et al., 2016; Proudfoot et al., 2017; Emwas et al., 2020; Mazzorana et al., 2020). They routinely also measure *k*_off_ values (Pollard, 2010). However, they cannot usually access the structural determinants of the transition states associated with ligand unbinding. This information would be crucial to eventually design ligands with longer residence times. In contrast, all-atom molecular simulations (in particular molecular dynamics (MD)) can provide a detailed map of protein-ligand interactions and the atomic rearrangements that drive ligand unbinding. However, the residence time of tight binders can be as long as several hours (Li et al., 2014), much longer than the timescales reached by plain MD (milliseconds on dedicated, specialized machines) (Pan et al., 2019; Shaw et al., 2021). Thus, *k*_off_ predictions based on such a straightforward approach so far have been few in number (Pan et al., 2017) or limited to model systems (Tang and Chang, 2018).

Enhanced sampling is a more general approach to the estimation of *k*_off_, regardless of the timescale of the unbinding event. One group of methods (including metadynamics, Gaussian Accelerated MD, scaled MD, and dissipation-corrected targeted MD**)** employs biasing potentials designed to reduce the free energy barrier determining the frequency of dissociations. Because the bias affects the dynamics, correction terms are required to recover the unbiased *k*_off_ from the biased rates. A second group is represented by path sampling approaches such as weighted ensemble and milestoning. These rigorously generate an ensemble of trajectories by iteratively restarting the (unbiased) simulations from selected configurations (typically closer to the transition state than expected from the equilibrium distribution) with the aim of increasing the likelihood of observing dissociations. Finally, Markov state models (MSMs) can provide a complete picture of the metastable states of the system and transition rates between them by analyzing molecular simulation data.

In this review, we summarize principles and applications of the three approaches outlined above (Sections 2–4). Next, we discuss the impact of force fields on the accuracy of the calculations (Section 5). Finally, we provide a perspective on how machine learning, along with exascale computing, could constitute one way to address these challenges (Section 6).

### 1.1 Scope

Many methods have been developed for the calculation of rate constants in biomolecular simulations. Here, we review methodologies that have been applied to the calculation of binding dissociation rates (*k*_off_) of protein-ligand complexes with a focus on the effect of the potential energy function. In particular, for the sake of conciseness, we do not cover methods that have been applied only to other types of systems/problems (e.g., supramolecular host-guest dissociations, peptide folding rates) and methods that enable relative comparisons of *k*_off_ between different ligands.^1^ For these methods, we refer the reader to the other excellent resources on the topic (Chong et al., 2017; Bruce et al., 2018; Nunes-Alves et al., 2020).

## 2 Biased MD Methods

In this class of methods, the system is biased (by adding a potential term to the Hamiltonian, or adding external forces) to favor the observation of unbinding events. The bias is designed to enhance the exploration along low-dimensional collective variables (CV), which are represented as differentiable functions *s*(x*)* of the atomic coordinates x. These describe the slow degrees of freedom governing the unbinding process. The CV must be able to distinguish the metastable states involved in the process i.e., configurations in different states should correspond to different values of the CV. The identification of optimal CVs (whenever they are not intrinsic in the technique) is a complicated task, and their identification is at the center of a heated debate that is still open (Sittel and Stock, 2018). Because biasing terms alter the dynamics, methods which recover the kinetic parameters of the unbiased system from its free energy surface have been devised. The majority of biased methods adopt specific corrections based on Kramers’ rate theory

$$k_{AB}\;=\;\omega_{A}\kappa_{A}\frac{Z^{*}}{Z_{A}}\;$$ (1)

where *k*_*AB*_ is the rate of transition from state A to B (in this case the bound and unbound states), $\omega_{A}$ is typically associated with the curvature of the free energy surface, $\kappa_{A}$ is the transmission coefficient, and $Z^{*}$ and $Z_{A}$ are the configurational partition functions of the transition state and state A, respectively. These methods require the identification of the transition state ensemble, defined as the set of conformations of highest free energy along the (un)binding pathway. This is in general a challenging task for drug binding processes, which can involve multiple dissociation pathways due to the conformational flexibility of the protein (Plattner and Noé, 2015). Approaches of this kind have been developed for Gaussian accelerated molecular dynamics (Miao et al., 2020) (see Section 2.1), dissipation-corrected Targeted Molecular Dynamics (Wolf and Stock, 2018) (see Section 2.2), and *τ*-random acceleration molecular dynamics (Kokh et al., 2018) (see Section 2.3)^2^. If no bias is deposited on the region of the transition state(s), the kinetic correction can be assumed not to depend on $\kappa_{A}$ and $Z^{*}$ (Voter and Doll, 1985; Hänggi et al., 1990; Truhlar et al., 1996). This simplifies dramatically the rate estimation, and it is used for ligand unbinding in the kinetics-oriented flavors of metadynamics (Tiwary and Parrinello, 2013; Wang et al., 2018) (see Section 2.4).

### 2.1 Ligand Gaussian Accelerated MD

#### 2.1.1 Basic principles

**FIGURE 1:**
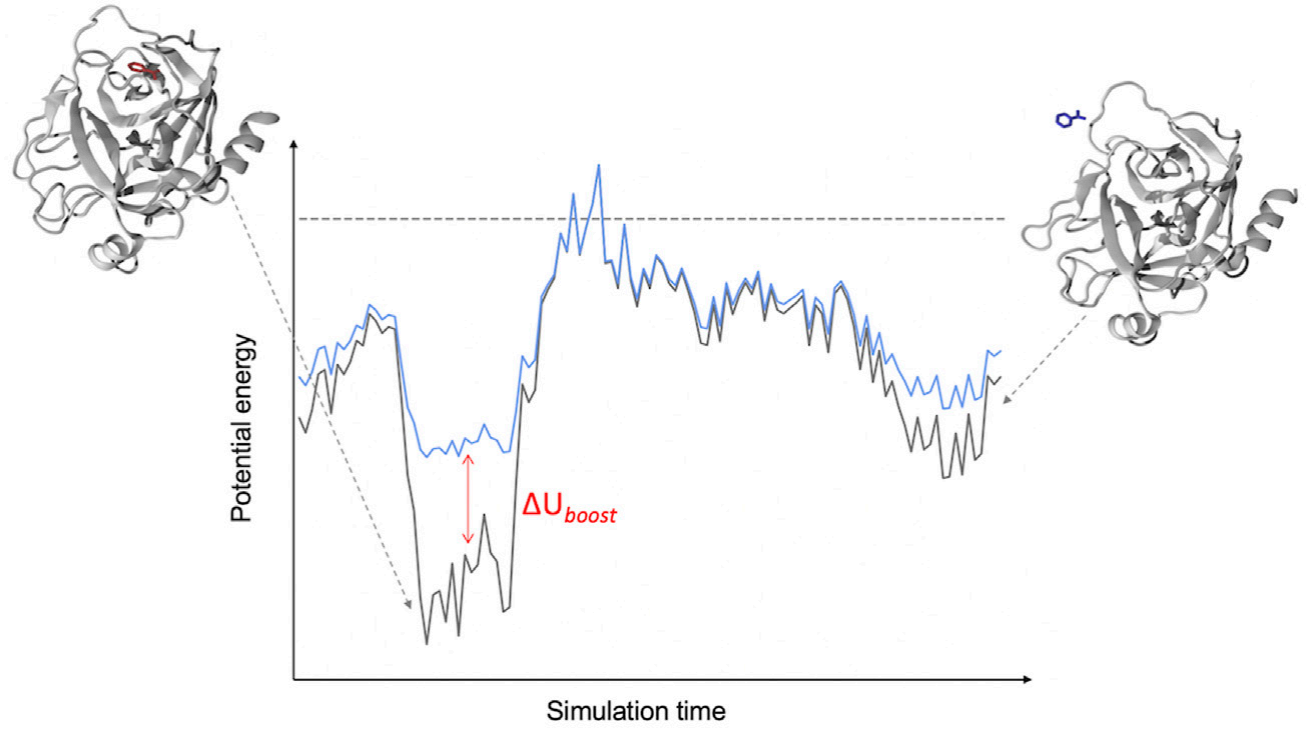
Schematic of a LiGaMD Simulation. The LiGaMD potential (∆U_boost_) acts when the potential energy of a protein-ligand complex (black line) is below a predefined threshold (dashed line), adding a harmonic potential to raise the energy of the system (cyan line) and favor the exploration of the conformational space of the ligand-protein complex.

In this approach (Miao, 2018), two harmonic potentials are added to the non-bonded component of the potential energy so as to lower the binding/unbinding free energy barrier (Figure 1). These potentials act on the following CVs: 1) the ligand-environment potential energy and (optionally) 2) the rest of the system potential energy. Both biasing potentials are capped at user-defined thresholds. Computing the correction to recover the unbiased transition rate requires the estimation of the potential of mean force (PMF) profile and free energy barrier as a function of a separate CV describing the binding process e.g., a distance between ligand and protein atoms (Miao, 2018). In the closely related Pep-GaMD method, developed specifically for simulating peptides unbinding from their target protein, the harmonic “boost” potentials are applied to the total potential (both non-bonded and bonded components) along the CVs (Wang and Miao, 2020). The application of the additional boost potential to the bonded component of the peptide potential energy accelerates the sampling of its conformational flexibility.

#### 2.1.2 Applications

So far, the approach has been successfully applied to the ligand benzamidine targeting the trypsin enzyme (Miao, Bhattarai and Wang, 2020), using the AMBER14SB (Maier et al., 2015) and GAFF (Wang et al., 2004) force-fields. The calculated *k*_off_ = 3.53 ± 1.41 s^−1^ was two orders of magnitude smaller than the experimental value of 600 ± 300 s^−1^ (Guillian and Thusias, 1970). The simulations required a cumulative 5 μs of MD for the estimation of *k*_off_. Pep-GaMD has been used to investigate the un/binding of three model peptides that target the SH3 domain—one of which (PDB:1CKB) has an experimentally determined *k*_off_ available for comparison to the computed value. Employing the AMBER14SB (Maier et al., 2015) force field and an aggregate simulation time of 3 μs, a *k*_off_ of 1.45 ± 1.17 **⋅** 10^3^ s^−1^ was computed for 1CKB; a result that is in close agreement with the experimental value of 8.9 **⋅** 10^3^ s^−1^ (Xue et al., 2014).

### 2.2 Dissipation-Corrected Targeted Molecular Dynamics (dcTMD)

#### 2.2.1 Basic principles

This method (Wolf and Stock, 2018) assumes that unbinding processes (along with binding processes) can be described by the 1-dimensional Langevin dynamics of a suitable CV. The approach requires the determination of the free energy profile and the Langevin friction coefficient as a function of such a CV. These can be calculated from a nonequilibrium targeted molecular dynamics simulation (Schlitter et al., 1994) (see Supplementary Material S4), where a pulling force drives the system at a constant speed along the CV. Dissociation rates could then be obtained by performing the unbiased 1-dimensional Langevin dynamics simulations (Wolf and Stock, 2018). Despite the simplification, the timescales of ligand unbinding processes at room temperature still lead to prohibitively expensive simulations. To tackle this problem, the authors later introduced an approach that uses Kramers’ theory to correct the rates obtained from Langevin simulations performed at higher temperatures. (Wolf et al., 2020).

#### 2.2.2 Applications

The method has been successfully applied to the calculation of *k*_off_ of the trypsin-benzamidine complex, and the complex between a resorcinol scaffold-based inhibitor and the HSP90 protein. The calculated values 270 ± 40 s^−1^ and 1.6 ± 0.2 **⋅** 10^–3^ s^−1^ respectively, agree well with the experimental values of 600 ± 300 s^−1^ (Guillian and Thusias, 1970) and 3.4 ± 0.2 **⋅** 10^–2^ s^−1^ (Amaral et al., 2017) These predictions required an aggregate of ∼ 1.5 × 10^4^ ms of Langevin simulations and used the AMBER99SB* force-field (Best and Hummer, 2009).

### 2.3 *τ*RAMD

#### 2.3.1 Basic principles

The *τ*-random acceleration molecular dynamics (*τ*RAMD) (Kokh et al., 2018) protocol is a quasi-biased method that evolved from random acceleration molecular dynamics (RAMD) (Lüdemann et al., 2000). *τ*RAMD simulations of ligand-protein systems proceed similarly to standard MD simulations, without the need for any prior parameter fitting, characterization of CVs or binding pathways. The user specifies the magnitude of a randomly oriented force that is applied to the ligand to accelerate its dissociation from the binding pocket at each checkpoint, allowing for the observation of dissociation pathways within several nanoseconds of simulation time. The magnitude of the force dictates the duration of simulation time that is required and is reported to have a minimal effect on the accuracy of computed residence times. The direction of the force is reassigned after each checkpoint until the ligand COM moves past a certain distance threshold from its previous position. If the deviation of the ligand COM meets or exceeds this threshold after the force is applied, the direction of the force is retained until the following checkpoint. An ensemble of unbinding simulations is spawned from different starting configurations and velocities, and the ensemble-averaged residence time is calculated from the bootstrapped distribution of the time taken for dissociation to occur.

#### 2.3.2 Applications

The earliest applications of *τ*RAMD for unbinding kinetics focused on qualitatively ranking ligands according to their computed *k*_off_ values (see Supplementary Material S5) (Kokh et al., 2018, 2019, 2020). Recently, the first quantitative *τ*RAMD application was demonstrated by Maximova and co-workers (Maximova et al., 2021), who formulated a Kramers’ rate theory-based rescaling factor to correct for the influence of the applied force on the receptor-ligand coupling (which previously limited the method to qualitative ranking) to obtain a quantitative *k*_off_ estimate for the drug Isoniazid unbinding from the catalase enzyme. Using seven trajectories (with applied forces of different magnitudes), and the CHARMM36 forcefield (Best et al., 2012), a *k*_off_ of 2.8 ± 3.7 **⋅** 10^–2^ s^−1^ was computed—a result which agreed very well with the experimental equivalent of 2.0 ± 0.3 **⋅** 10^–2^ (Singh et al., 2008).

### 2.4 Metadynamics-Derived Methods

#### 2.4.1 Basic principles

**FIGURE 2:**
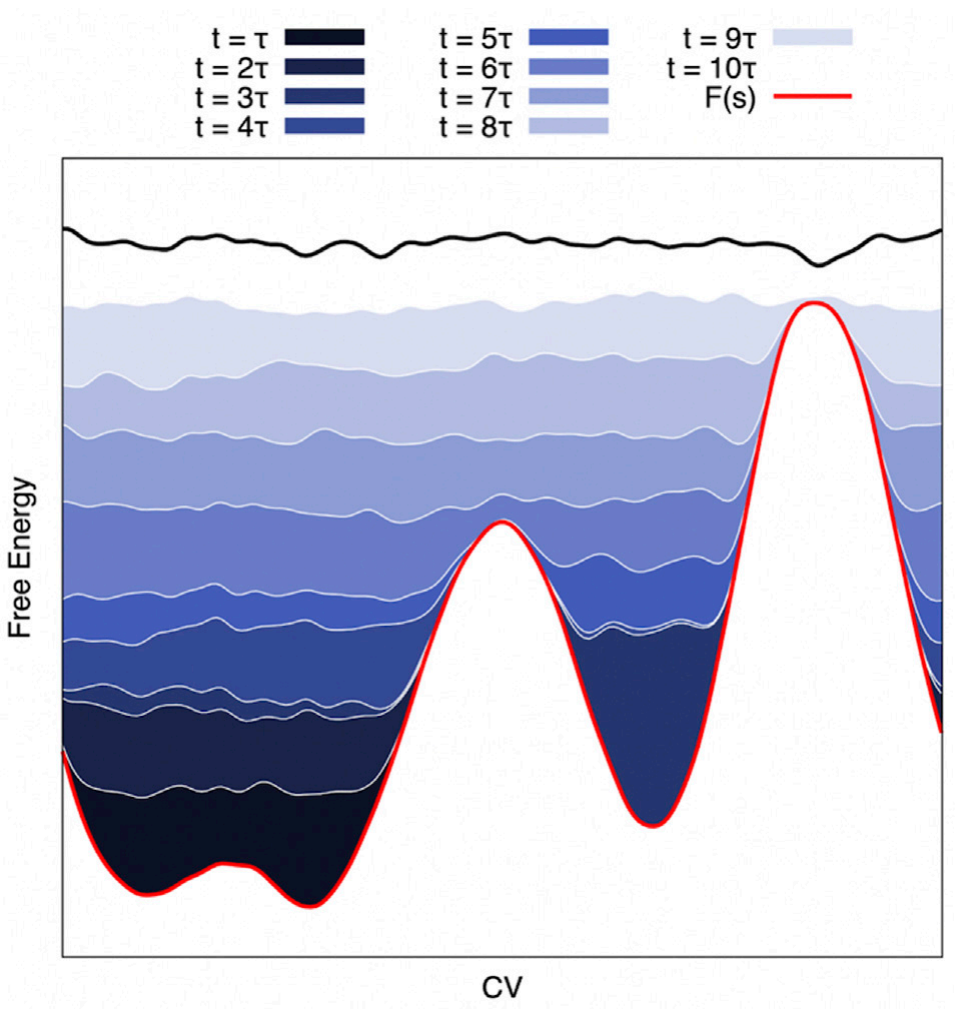
Schematic of a Metadynamics Simulation. On the CV-projected FES (red line), MetaD deposits a series of gaussians that sum up (from dark blue to white) until the system becomes diffusive in the CV space. This approach can be exploited to reduce the barrier height to have a reasonable transition time and reweight it a posteriori for an estimation of the kinetic constants (see Text).

Well-tempered Metadynamics (MetaD) (Laio and Parrinello, 2002) is an exact free-energy method (Barducci et al., 2008; Dama et al., 2014). It draws inspiration from earlier CV-based enhanced sampling techniques such as local elevation (Huber et al., 1994), Wang-Landau (Wang and Landau, 2001), conformational flooding (Grubmüller, 1995), and adaptive umbrella sampling techniques (Hooft et al., 1992; Bartels and Karplus, 1997). In MetaD, a history-dependent bias potential *B*_*t*_(*s*) is built iteratively by adding Gaussian functions (as approximations of CV histograms) to the potential at regular intervals throughout the simulations. Several different bias-deposition schemes have been devised (Bussi and Laio, 2020). Ultimately, convergence is achieved when the sum of the free energy surface and the bias potential produces a flat landscape that results in diffusive dynamics in CV space (see Figure 2). It is then possible to compute the free energy surface along the CV *via* reweighting methods, such as Weighted Histogram Analysis Method (WHAM) (Kumar et al., 1992), Multistate Bennet Acceptance Ratio (MBAR) (Shirts and Chodera, 2008), or other estimators (Tiwary and Parrinello, 2015; Schäfer and Settanni, 2020).

MetaD has been extended to allow recovery of the kinetics of the unbiased ensemble. The method speeds up the calculation of kinetic rates by filling up the starting free energy basin so as to reduce the activation free energy barrier to ∼ *k*_B_T. This way, the biased residence time of the system in the initial state is small enough to allow multiple observations of the transition. Transition times obtained in the biased ensemble are then scaled to recover the unbiased kinetics. Following the approaches of Grubmüller (Conformational flooding (Grubmüller, 1995)) and Voter (Hyperdynamics (Voter, 1997)) the unbiased transition time is connected to the biased time by:

$$t_{\text{unbiased}}=\text{α}\;t_{\text{biased}}$$

$$=\overset{t_{\text{biased}}}{\underset{t=0}{\sum }}\exp\left(\beta B_{t}\left(s\left(t\right)\right)\right)\text{Δ}t$$ (2)

where *β* =(*k*_B_T)^−1^, *Bt(s)* is the history-dependent bias potential, and Δ*t* is the time step. For this last equation to be valid, no bias should be present on the transition state. In the so-called infrequent MetaD variant (Tiwary and Parrinello, 2013), the Gaussians are deposited less frequently in barrier regions than they are in standard MetaD, thus lowering the probability of adding bias to the transition state. In frequency-adaptive (FA) MetaD (Wang et al., 2018), the time interval between bias depositions is gradually increased as the system approaches the transition state. After an initial fast filling of the free energy minimum, the same deposition rate as infrequent MetaD is achieved. This way, results are obtained at a lower computational cost compared to standard infrequent MetaD. Recently, an alternative method to infrequent and frequency-adapted MetaD has been presented. (Ansari et al., 2022) This method builds on a variant of MetaD called on-the-fly probability enhanced sampling (OPES). (Invernizzi and Parrinello, 2020) In the new approach, called OPES-flooding, the bias is constructed in a fast but controlled manner to fill the starting metastable basin up to a user-defined threshold value to automatically avoid depositing bias on the transition state. Usually, the standard protocol adopted in infrequent, FA-MetaD and OPES-flooding consists of running multiple independent simulations that yield an empirical distribution of residence times. A statistical analysis based on the Kolmogorov-Smirnov (KS) test (Salvalaglio et al., 2014), details in Supplementary Material S1) is then used to verify *a posteriori* that the transition state was indeed untainted.

#### 2.4.2 Applications

Infrequent MetaD simulations based on the OPLS force-field (Kaminski et al., 2001) were used to study the unbinding of the ligand dasatinib from its target c-Src kinase (Tiwary et al., 2017). The CVs were the distance between the ligand and the binding pocket and the solvation state of the binding pocket. The calculated *k*_off_ of 4.8 ± 2.4 ·10^–2^ s^−1^ of dasatinib to c-Src obtained from 12 unbinding trajectories agreed well with an experimental value (5.6 · 10^–2^ s^−1^, measured indirectly from *k*_on_) published by (Shan et al., 2009), but differed from a second value obtained for a fluorophore-tagged analogue (1.8 · 10^–4^ to 7.9 **⋅** 10^–4^ s^−1^) (Kwarcinski et al., 2016). A similar protocol was used to calculate *k*_off_ for 1-(3-(tert-butyl)-1-(p-tolyl)-1H-pyrazol-5-yl)urea), an inhibitor of p38 MAP II kinase belonging to the BIRB-796 family, this time using AMBER99SB-ILDN (Hornak et al., 2006; Lindorff-Larsen et al., 2010) and GAFF force-fields (Wang et al., 2004; Wang J. et al., 2006). After 17 independent unbinding events, the calculated *k*_off_ (0.020 ± 0.011 s^−1^ (Casasnovas et al., 2017)) was almost one order of magnitude lower than the experimental value of 0.14 s^−1^ (Regan et al., 2003). Two other CVs yielded very similar results simulating 10 unbinding events each, suggesting that the discrepancy between the calculated and experimental values most likely arises from uncertainty in the force field rather than the choice of CVs.

FA-MetaD and Infrequent MetaD were used by Wang and co-authors (Wang et al., 2018) to obtain *k*_off_ values for benzene and indole ligands from the binding pocket of the L99A mutant of T4 lysozyme using CHARMM22 (MacKerell et al., 1998; MacKerell et al., 2004) and CGenFF (Vanommeslaeghe et al., 2011). The calculated *k*_off_ for benzene lay within the range of 4–10 s^−1^, around 100-fold lower than the experimental value of 950 ± 20 s^−1^ (Feher et al., 1996). Both MetaD protocols used the same force-field, sample size (20 replicas), and path-CVs (Branduardi et al., 2007; Wang et al., 2017). CHARMM36-based (Best et al., 2012) infrequent MetaD simulations (Mondal et al., 2018) yielded a *k*_off_ for benzene (270 ± 100 s^−1^) that was considerably closer to the experimental value. Although only the displacement between binding pocket and ligand centers-of-mass was used as the CV, and the sample size was smaller than that of the previous study by Wang et al, it is tempting to conclude that even a different version of the same force-field (CHARMM in this case) may significantly impact the result.

More recently, AMBER14SB-based (Maier et al., 2015) FA-MetaD simulations were applied to study the unbinding kinetics of a radioligand, iperoxo, from the M_2_ human muscarinic acetylcholine receptor (Capelli et al., 2020). The calculated *k*_off_ (3.7 ± 0.7 **⋅** 10^−4^ s^−1^) was two orders of magnitude smaller than the experimental value (1.0 ± 0.2 **⋅** 10^−2^ s^−1^). Density Functional Theory (DFT)-based QM/MM calculations suggested that this estimation discrepancy may be ascribed, at least in part, to the lack of polarization and charge transfer effects lacking in standard biomolecular force fields (Capelli et al., 2020).

OPES-flooding simulations based on AMBER14SB (Maier et al., 2015) and GAFF (Wang et al., 2004) were recently applied to study the unbinding kinetics of the trypsin-benzamidine complex, unveiling the role of water in regulating the residence time. Notably, the authors identified two different unbinding pathways and were able to calculate the corresponding rates separately. The slowest rate of 687 s^−1^ that is supposed to dominate the residence time is in good agreement with the experimental value of 600 ± 300 s^−1^ (Guillian and Thusias, 1970).

## 3 Markov State Models

### 3.1 Basic Principles

**FIGURE 3:**
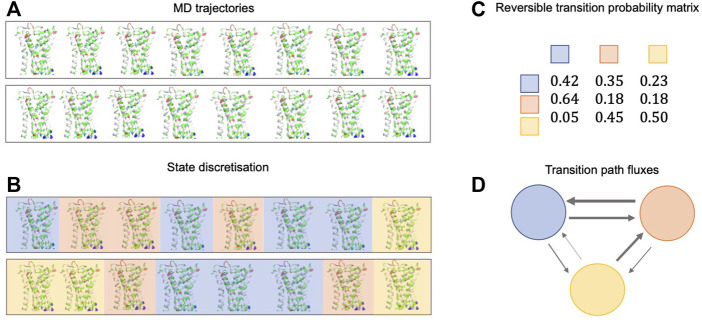
Simplified schematic depiction of the MSM construction pipeline. **(A)** Several continuous MD trajectories are simulated in parallel. **(B)** The trajectories are discretized. **(C)** A reversible transition probability matrix is calculated from a matrix of state-to-state transition counts **(D)** Probability fluxes between states (gray arrows, with line thickness representing the magnitude of the flux) indicate the highest likelihood transition paths and can be used to calculate the mean first passage time (MFPT) between states.

Markov state models (MSMs) (Singhal et al., 2004) are discrete models describing the dynamics of a system in terms of transition probabilities between a finite set of metastable states. The fundamental ingredients of the method are 1) a discretization of the conformational space into (kinetically fast) microstates and 2) a transition matrix that describes the probability of observing the system in another microstate after a fixed lag time *t.* An interpretable, coarse-grained model is then built by defining kinetically metastable macrostates as collections of microstates, and this model can provide *k*_off_ values. Figure 3 shows a simplified schematic depiction of the MSM construction pipeline. The lag time *t* must be long enough to ensure that transitions between states are approximately Markovian^4^ and short enough for the model to represent all relevant fast processes. It should be chosen to be faster than association events to avoid systematic overestimation of the residence time (Paul et al., 2017). When this is not possible, *k*_off_ can still be estimated from rate matrices rather than transition matrices (Kalbfleisch and Lawless, 1985; Crommelin and Vanden-Eijnden, 2009). However, rate matrix estimation is not unique and different approaches can result in residence times that differ even by an order of magnitude (Paul et al., 2017).

The input data to build MSMs can come from an ensemble of unbiased MD trajectories that sample dissociation events. However, generating this data is usually prohibitively expensive. Hence, several powerful schemes have been designed to enable the estimation of second-long residence times from relatively short MD simulations. These include adaptive restarting strategies (Bowman et al., 2010; Wan and Voelz, 2020) and/or biased simulations (Wu et al., 2016; Paul et al., 2017; Stelzl et al., 2017). In particular, recently developed estimators such as transition-based reweighting analysis TRAM (Wu et al., 2016) and its MBAR variant TRAMMBAR (Paul et al., 2017) require only irreversible visits to metastable states in the unbiased MD (as long as these states are sampled reversibly in the biased ones) and can greatly alleviate the sampling problem.

### 3.2 Applications

MSM calculations on the trypsin-benzamidine complex (Plattner and Noé, 2015) (methodological details in Supplementary Material S6) yielded a *k*_off_ of 131 ± 109 × 10^2^ s^−1^, which compares fairly well with experiments (*k*_off_ = 600 ± 300 s^−1^) (Guillian and Thusias, 1970). However, the high level of uncertainty suggests that sampling of unbinding events might be insufficient despite the large amount of aggregate simulation time (149.1 μs in this case). The dissociation of benzene from the L99A mutant T4 Lysozyme was investigated in a hybrid MSM/infrequent MetaD study (Mondal et al., 2018) using the CHARMM36 force-field (Best et al., 2012). The MSM was constructed from unbiased MD trajectories, and gave a *k*_off_ of 310 ± 130 s^−1^, which was marginally closer to the experimental *k*_off_ (950 ± 20 s^−1^) (Feher et al., 1996) than the value reported by the accompanying infrequent MetaD simulations (*k*_off_ = 270 ± 100 s^−1^) (Mondal et al., 2018) and considerably closer than the previous FA-MetaD-based predictions (see Table 1) (Wang et al., 2018) However, the statistical uncertainty in the MSM-derived *k*_off_ was quite large, and the calculation required more simulation time (60 μs) compared biased MD approaches to obtain similar uncertainties: FA-MetaD/Infrequent MetaD studies typically require 6–12 μs (Casasnovas et al., 2017; Wang et al., 2018; Capelli et al., 2020) and LiGaMD (Miao et al., 2020) required ∼ 5 μs.

**TABLE 1:** Quantitative in silico calculations (we highlighted in boldface the simulations that are below one order of magnitude for the predicted results with respect to the experimental ones)

Target	Technique	T [K]	Force field	*k*_off_ (sim) [s^−1^]	*k*_off_ (Exp) [s^−1^]	Simulation time [µs]	Ref	Year
Trypsin/Benzamidine	SEEKR	298	Amber14SB + GAFF	83 ± 14	600 ± 300	19	10.1021/acs.jpcb.6b09388	2017
Trypsin/Benzamidine	SEEKR	298	Amber14SB + GAFF	174 ± 9	600 ± 300	4.4	10.1021/acs.jctc.0c00495	2020
Trypsin/Benzamidine	SEEKR2	298	Amber14SB + GAFF	990 ± 70	600 ± 300	5	10.26434/chemrxiv-2021-pplfs	2021
Trypsin/Benzamidine	M-WEM	298	Amber14SB + GAFF	791 ± 197	600 ± 300	0.48	10.1021/acs.jctc.1c00803	2022
Trypsin/Benzamidine	Inf-MetaD	300	Amber99SB-ILDN	9.1 ± 2.5	600 ± 300	5	10.1073/pnas.1424461112	2015
Trypsin/Benzamidine	Inf-MetaD	300	Amber14SB + GAFF	4176 ± 324	600 ± 300	—	10.1021/acs.jctc.8b00934	2019
Trypsin/Benzamidine	MSM	298	Amber99SB + GAFF	(9.5 ± 3.3)·10^4^	600 ± 300	50	10.1073/pnas.1103547108	2011
Trypsin/Benzamidine	MSM	—	—	2.8 ·10^4^	600 ± 300	7.7	10.1021/ct400919u	2014
Trypsin/Benzamidine	MSM	—	Amber99SB + GAFF	131 ± 109	600 ± 300	149.1	10.1038/ncomms8653	2015
Trypsin/Benzamidine	MSM	298	Amber99SB + GAFF	1170 [617, 2120]	600 ± 300	58.28	10.1073/pnas.1525092113	2016
Trypsin/Benzamidine	WExplore	300	Charmm36 + CGenFF	5.56 ·10^4^	600 ± 300	4.1	10.1016/j.bpj.2017.01.006	2017
Trypsin/Benzamidine	REVO	300	Charmm36 + CGenFF	2660	600 ± 300	8.75	10.1063/1.5100521	2019
Trypsin/Benzamidine	LiGaMD	300	Amber14SB + GAFF	3.53 ± 1.41	600 ± 300	5	10.1021/acs.jctc.0c00395	2020
Trypsin/Benzamidine	dcTMD	290	Amber99SB*	270 ± 40	600 ± 300	10000^c^	10.1038/s41467-020-16655-1	2020
Trypsin/Benzamidine	AMS	298	Charmm36 + CGenFF	260 ± 240	600 ± 300	2.3	10.1021/acs.jctc.6b00277	2016
Trypsin/Benzamidine	OPES	300	Amber14SB + GAFF	687	600 ± 300	3.2	arXiv:2204.05572	2022
T4L L99A-Benzene	In-MetaD	300	Charmm22*	6.0 ± 2.2	950 ± 200^a^	6.7	10.1039/c7sc01627a	2017
T4L L99A-Benzene	FA-MetaD	300	Charmm22*	5.7 ± 2.3	950 ± 200^a^	5.5	10.1063/1.5024679	2018
T4L L99A-Benzene	In-MetaD	303	Charmm36	270 ± 100	950 ± 200	—	10.1371/journal.pcbi.1006180	2018
T4L L99A-Benzene	MSM	303	Charmm36	310 ± 130	950 ± 200	60	10.1371/journal.pcbi.1006180	2018
T4L L99A-Indole	In-MetaD	300	Charmm22* + CGenFF	9.8 ± 10.2	325 ± 75^b^	4.5	10.1063/1.5024679	2018
T4L L99A-Indole	FA-MetaD	300	Charmm22* + CGenFF	6.0 ± 3.7	325 ± 75^b^	2.0	10.1063/1.5024679	2018
µOpioid receptor-morphine	In-MetaD	300	Charmm36 + CGenFF	(5.7 ± 0.5)·10^–2^	(2.3 ± 0.2)·10^–2^	6	10.1063/5.0019100	2020
µOpioid receptor-bruprenorphine	In-MetaD	300	Charmm36 + CGenFF	(2.1 ± 0.3)·10^–2^	(1.8 ± 0.3)·10^–3^	19	10.1063/5.0019100	2020
µOpioid receptor-Fentanyl	In-MetaD	310	Charmm36m + CGenFF	(2.6 ± 0.8)·10^–2^ (HID) (3.8 ± 1.4)·10^–1^ (HIE) 1.1 ± 0.3 (HIP)	4.2 · 10^–3^	6	10.1021/jacsau.1c00341	2021
TSPO-PK11195	REVO	300	Charmm36 + CGenFF	(D1)6.4 · 10^–5^ (D2)6.67·10^1^ (D3)6.4 · 10^–3^ (D4)4.1 · 10^–3^ (4RYI)6.0 · 10^–4^ (D1-D4 different docked poses)	4.9 · 10^–4^	40	10.1016/j.bpj.2020.11.015	2021
c-Src kinase-dasatinib	In-MetaD	300	OPLS	(4.8 ± 2.4)·10^–2^	5.6 · 10^–2^ 1.1 · 10^–3^	∼7–8	10.1126/sciadv.1700014	2017
Src kinase - imatinib	TS-PPTIS	305	Amber99SB*-ILDN + GAFF (QM/MM)	0.026	0.11 ± 0.08	—	10.1021/acs.jctc.8b00687	2018
Epoxide Hydrolase-TPPU	WExplore	300	Charmm36 + CGenFF	2.4 · 10^–2^ [3.6 · 10^–3^ s^−1^, 4.4 · 10^–2^ s^−1^]	1.5 · 10^–3^	6	10.1021/jacs.7b08572	2018
p38 kinase/1-(3-(tert-butyl)-1- (p-tolyl)-1H-pyrazol-5-yl)urea	In-MetaD	300	Amber99SB-ILDN + GAFF	0.020 ± 0.011	0.14	6.8	10.1021/jacs.6b12950	2017
M2 muscarinic receptor/iperoxo	FA-MetaD	310	Amber14SB + GAFF	(3.7 ± 0.7)·10^–4^	(1.0 ± 0.2)·10^–2^	8	10.1021/acs.jpclett.0c00999	2020
HSP90-inhibitor	dcTMD	300	Amber99SB + GAFF	(1.6 ± 0.2)·10^–3^	(3.4 ± 0.2)·10^–2^	5000^c^	10.1038/s41467-020-16655-1	2020
Mdm2/PMI	MSM	300	Amber99SB-ILDN	0.125 [0.025, 0.66] 1.13 [0.48, 1.33] (Different rate matrix estimators)	0.037 [0.029, 0.04]	500	10.1038/s41467-017-01163-6	2017
Mdm2/p53	MSM	300	Amber99SB-ILDN-NMR	1.9·10^5^	2.1	831	10.1016/j.bpj.2017.07.009	2017
SH3 Domain—1CKB	Pep-GaMD	300	Amber14SB	(1.45 ± 1.17)·10^–3^	8.9 · 10^–3^	3	10.1063/5.0021399	2020
MtKatG—Isonazid	τRAMD + extrapolation	300	CHARMM36 + SwissParam	(2.8 ± 3.7)·10^–2^	(2.0 ± 0.3)·10^–2^	—	10.1021/acs.jpclett.1c02952	2021

a The Authors in the original work considered the experimental k_off_ at 293 K (800 ± 200 s^−1^), while they simulated the system at 300 K. Here we choose to put the value at the closest temperature available in experiments (303K—950 ± 200 s^−1^). Both the experimental values come from (Feher et al., 1996).

b The experimental value has been measured at 293 K.

c For dcTMD, computational time is referred to 1D Langevin simulator, and the authors says that “1 ms of simulation time at a 5 fs time step take ∼6 h of wall-clock time on a single CPU”.

The use of biased simulations can greatly reduce the sampling requirements. Wu et al., (2016) showed that by integrating unbiased MD with umbrella sampling simulation data, only 5%–10% of the unbiased data was necessary to estimate the dissociation rate of the trypsin-benzamidine complex up to statistical significance (*k*_off_ = 1170s^−1^ [617s^−1^, 2120s^−1^]). A combination of 500 μs of unbiased MD and 1 μs of Hamiltonian replica exchange simulation was used to create an MSM model describing the binding of the oncoprotein fragment Mdm2 and a peptide inhibitor PMI. Estimates based on two different post-processing schemes yielded values of *k*_off_ = 0.125 s^−1^ [0.025 s^−1^, 0.66 s^−1^] and *k*_off_ = 1.13 s^−1^ [0.48 s^−1^, 1.33 s^−1^], corresponding to a 10–30-fold overestimation relative to experiments (*k*_off_ = 0.037 s^−1^ [0.029 s^−1^, 0.04 s^−1^]) (Paul et al., 2017).

## 4 Path Sampling Methods

Path sampling methods focus on generating an ensemble of transition pathways between bound and unbound states. Typically, this class of methods accelerates the unbinding event by exploiting restarting strategies to favor the sampling of short trajectories in the vicinity of the transition state, which are then used to reconstruct the full unbinding process. Weighted Ensemble (WE) (Huber and Kim, 1996), milestoning (Cho et al., 2006; Elber, 2007), transition state-partial path interface sampling (TS-PPTIS) (Juraszek et al., 2013), and adaptive multilevel splitting (AMS) (Cérou and Guyader, 2007; Cérou et al., 2011) are path sampling methods that were employed in calculations of *k*_off_ for ligand/protein complexes.

### 4.1 Weighted Ensemble Methods

#### 4.1.1 Basic principles

A set of unbiased molecular dynamics trajectories with equivalent statistical weights are spawned in parallel from a ligand/protein complex in the ground-state configuration (Huber and Kim, 1996). The configuration space is then subdivided into bins, which the trajectories/walkers navigate through. The weighted ensemble (WE) method aims to maintain a fixed number (*N*) of walkers per bin. Thus, the occupancy of the bins is calculated at specific resampling intervals τ_int_. If the number of walkers in a given bin is lower than *N,* one or more of the walkers are cloned, with each daughter trajectory receiving a fraction of the weight of the original. Conversely, in regions populated by a number of walkers exceeding *N,* two or more trajectories are merged, with the resulting trajectory inheriting the weights of its constituents (Zuckerman and Chong, 2017). This process results in a resampled trajectory space spanning the bound, intermediate and unbound states from which *k*_off_ values can be obtained (Zhang et al., 2010).

Notably, the method does not require a detailed a prori definition of differentiable collective variables, and it is embarrassingly parallel. Given that the availability of Tier-0 and Tier-1 machines has grown significantly since the method was first formulated, several scalable open-source implementations have emerged. These include WExplore (Dickson and Brooks, 2014), Wepy (Lotz and Dickson, 2020), and REVO (Resampling of Ensembles by Variation Optimization) (Donyapour et al., 2019). The latter is a method featuring a novel resampling algorithm replacing bins in configurational space with a system-specific all-to-all pairwise distance matrix between walkers, thereby decreasing the correlation between trajectories. The novel concurrent adaptive sampling (CAS) algorithm (Ahn et al., 2017) builds on the traditional WE method by adaptively constructing macrostates (represented by *n*-dimensional Voronoi cells) while approximating the committor function of each macrostate, and clustering the macrostates according to their committor functions as the simulation progresses. This improves the efficiency of WE simulations in high-dimensional systems, by directing computational power to sampling portions of configuration space that are closer to the “product” configuration.

#### 4.1.2 Applications

The *k*_off_ of the trypsin-benzamidine complex as calculated by WExplore (5560 s^−1^) (Dickson and Lotz, 2017) overestimated by one order of magnitude the experimental value (600 s^−1^) (Guillian and Thusias, 1970). This value was calculated from five independent WExplore runs, corresponding to an aggregate simulation time of 4.1 μs. Using clustering-based confirmation space network analysis techniques (Dickson and Brooks, 2013), three distinct ligand exit pathways were unearthed from the trajectories. The trypsin-benzamidine system was later investigated again with REVO (Donyapour et al., 2019). Based on five independent REVO runs, giving a total of 8.75 μs, a *k*_off_ of 2660 s^−1^ was predicted—a minor improvement on WExplore, but an overestimation nonetheless. WExplore was also employed to estimate the dissociation rate of the TPPU inhibitor from soluble epoxide hydrolase. The calculated *k*_off_ (2.4 **⋅** 10^–2^ s^−1^ [3.6 **⋅** 10^–3^ s^−1^, 4.4 **⋅** 10^–2^ s^−1^]) was one order of magnitude greater than the experimental value of 3.6 **⋅** 10^–3^ s^−1^. (Lotz and Dickson, 2018), and required 6 μs of simulation time to compute. However, the reason for the systematic overestimations of *k*_off_ is not explicitly addressed. REVO was recently employed (Dixon et al., 2021) to quantify *k*_off_ values for five distinct bound poses of the PK-11195 radioligand in complex with TSPO (see Table 1), using a cumulative 5.18 μs of simulation time per pose. The calculated values for the poses spanned five orders of magnitude, and the pose with the most favorable docking score (pose D1, *k*_off_ = 6.4 × 10^–5^ s^−1^) exhibited the closest agreement with the experimental value (4.9 × 10^–4^ s^−1^) out of all the docked poses. All the of the studies described here made use of the CHARMM36 (Best et al., 2012) and CGenFF (Vanommeslaeghe et al., 2011) force fields. At present, the CAS method described in Section 4.1.1 has been successfully applied to host-guest systems only (Ahn et al., 2020).

### 4.2 Milestoning

#### 4.2.1 Basic principles

Here, the configuration space is treated as a coarse mesh characterized by slowly relaxing variables, such as native contacts and/or distances between chemical groups that describe the ligand unbinding process (Cho et al., 2006; Elber, 2007). The mesh must be coarse enough for distinct long-lived metastable states to emerge, but fine enough to ensure that transitions between the interfaces between the mesh’s cells or “milestones” are accessible in MD simulations. Equilibrium configurations for each milestone are usually generated with “pulling” SMD simulations, or with a series of MD simulations in which the diffusing group is harmonically restrained to the milestone surface. Afterward, a set of trajectories is spawned from each milestone, and whenever a trajectory reaches a new neighboring milestone, it is terminated. In practice, the criteria for termination of trajectories vary depending on the implementation. The lifetime and flux (i.e., number of trajectories passing through the milestone per unit time) associated with each milestone may be used to compute the ligand residence time (Elber, 2020).

Practical implementations of milestoning in ligand unbinding studies fall into two categories: 1) The Simulation Enabled Estimation of Kinetic Rates (SEEKR) (Votapka et al., 2017) approach, which exploits milestoning theory in a multiscale framework based on MD and Brownian Dynamics (BD) simulations (Luty et al., 1993). The milestones are nested spherical shells surrounding the binding pocket. Transitions between milestones close to the binding pocket are simulated using all-atom MD. Meanwhile, transitions between the more diffuse milestones further away are described by cheaper BD simulations—where fast sampling of rigid body interactions is more important than detailed sampling of ligand conformations. An updated implementation of SEEKR, named MMVT SEEKR, has been subsequently proposed (Jagger et al., 2020): it circumvents the need to compute the equilibrium distribution for all the milestones, reducing the computational time needed to compute kinetics constants. 2) The recently formulated weighted ensemble milestoning (WEM) methods combine milestoning theory with WE methods. Here, the configurational space between the milestones is split into bins, and WE simulations are run in between milestones to achieve faster convergence (Ray and Andricioaei, 2020).

#### 4.2.2 Applications

All applications to kinetics of biological systems so far are based on AMBER14SB (Maier et al., 2015) and GAFF (Wang et al., 2004) force fields and applied to the trypsin-benzamidine complex. SEEKR yielded a *k*_off_ of 83 ± 14 s^−1^ for the trypsin-benzamidine system using 19 μs of aggregate MD and ten spherical milestones. These results underestimate the experimental value (600 ± 300 s^−1^) (Guillian and Thusias, 1970). MMVT SEEKR improved the *k*_off_ estimate (174 ± 9 s^−1^), with only a quarter of the aggregate simulation time (4.4 μs) used in the prior SEEKR study. WEM (Ray and Andricioaei, 2020) gave a further improvement *k*_off_ = 791 ± 197 s^−1^, using a mere 0.5 μs of simulation time (Ray et al., 2022).

### 4.3 Transition State-Partial Path Transition Interface Sampling

#### 4.3.1 Basic principles

In transition state-partial path transition interface sampling (TS-PPTIS) (Juraszek et al., 2013) an initial metadynamics calculation is performed to determine the transition state and the free energy barrier along a given CV. Then, the transmission coefficient is estimated, similarly to the PPTIS method (Van Erp et al., 2003; Moroni et al., 2004) by foliating the barrier region along the CV with interfaces and sampling short trajectories spanning three consecutive interfaces. These trajectories are sampled using transition path sampling (Pratt, 1986; Dellago et al., 1998). Under the assumptions that the dynamics in the barrier region is diffusive and there are no memory effects for travelled distances beyond two interfaces, the kinetic rates are independent of the CV.

#### 4.3.2 Applications

TS-PPTIS was used to compute the *k*_off_ of the imatinib-Src kinase complex (Morando et al., 2016). The calculation used 5 CVs: 2 path collective variables (Branduardi et al., 2007), a CV counting the number of water molecules interacting with the ligand and the binding cavity, and two distances between key residues of Src characterizing the motion of the kinase A-loop. Using AMBER99SB*-ILDN and GAFF, the authors computed a value of *k*_off_ = 0.0114 s^−1^ [0.001 s^−1^, 0.139 s^−1^], which is slow (but within statistical significance) compared to experiments (*k*_off_ = 0.11 ± 0.08 s^−1^). In a separate work (Haldar et al., 2018), the authors refined the prediction by computing a free energy correction from the MM to a hybrid quantum mechanics/molecular mechanics Hamiltonian using a replica exchange thermodynamic integration scheme (Woods et al., 2003) and Metropolis-Hastings Monte Carlo sampling (Woods et al., 2008). This correction does not account for dynamical effects but only for changes in the free energy. The computed correction to *k*_off_ was small but consistent with faster dissociation dynamics obtaining a corrected value of *k*_off_ = 0.026 s^−1^.

### 4.4 Adaptive Multilevel Splitting

#### 4.4.1 Basic principles

Similarly to WE, adaptive multilevel splitting (AMS) (Cérou and Guyader, 2007; Cérou et al., 2011) relies on a set of trajectories that are systematically cloned or killed. However, AMS does not require bins. Instead, the algorithm is initialized by generating a set of “loop” trajectories starting and ending in the bound state. At each iteration, the replica that travelled the least distance *d* from the bound state (measured through a CV) is killed, and a new loop is created by restarting a simulation from a point at the same distance *d* previously visited by one of the remaining replicas. This is repeated until all loops travelled a distance above a threshold value (defining the unbound state) before returning to the bound state. The dissociation rate is then estimated from this collection of trajectories.

#### 4.4.2 Applications

AMS was used to calculate the dissociation rate of trypsin-benzamidine using the CHARMM36 force field (Best et al., 2012) for trypsin and CGenFF (Vanommeslaeghe et al., 2011) for the ligand (Teo et al., 2016). As the CV, the authors used the distance between the center of mass of benzamidine and the alpha carbons of the amino acids close to the binding site. A suitable value for the threshold value of the CV was obtained through a steered MD simulation. Furthermore, 130 ns unbiased MD simulation was run to estimate the average time of a looping trajectory under the assumption that the short loops thus sampled represented the large majority of loops and thus dominated the average loop duration. In total, 2.3 μs of simulations were used to obtain a *k*_off_ = 260 s^−1^ ± 240 s^−1^, in good agreement with experimental measurements.

## 5 Limitations Associated With Force Fields

Table 1 summarizes the *k*_off_ predictions of various ligand/protein systems obtained using the methods discussed in previous sections. For completeness, we also report the temperature, total simulation time, and force field used. In about one-third of the cases, spanning all different classes of methodologies and force fields, the theoretical predictions are in the same order of magnitude as the experimental values, and in a few cases (shown in boldface in Table 1) reproduce them within statistical error. In most cases, however, calculated values show discrepancies from 1 to 2 orders of magnitude, regardless of the method and force field. Similarly, the only predictive study reported so far (Paul et al., 2017) reports values with an error of 1–2 orders of magnitude (albeit with large statistical errors) relative to experimental data performed afterwards.^5^ All these results, taken together, lead us to suggest that regular force fields may be, at times, not accurate enough to predict *k*_off_ values.

Determining the source of the observed errors is a difficult task without dedicated studies as the accuracy of the predictions depends on methodological aspects, sampling accuracy, and the potential energy function, which are subject to mutual cancellation (or amplification) of error. In this and the next section, we discuss the literature focusing on the effect of the potential.

### 5.1 Force Field Dependence of the Results

The published data indicate that careful parametrization of the force fields is essential to obtain *k*_off_ predictions. Comparison between the results obtained from brute force MD calculations on a set of ligands binding to a *β*-cyclodextrin (βCD) host showed that *k*_off_ predictions parametrizing βCD with the Q4MD force field (Cézard et al., 2011) were consistently more accurate (within one order of magnitude of experimental values) than the GAFF-based (Wang et al., 2004) estimates (Tang and Chang, 2018). On the other hand, the *k*_on_ estimates were consistently better for the GAFF model, which points to the difficulty of obtaining transferable potentials. In the case of benzene unbinding from L99A T4 lysozyme, infrequent MetaD simulations using CHARMM22 (MacKerell et al., 1998; MacKerell Jr. et al., 2004) yielded a significantly underestimated *k*_off_ in the range of 4–10 s^−1^ (Wang et al., 2018), while the same method combined with CHARMM36 (Best et al., 2012) produced a *k*_off_ (270 ± 100 s^−1^) (Mondal et al., 2018) considerably closer to the experimental value of 950 ± 20 s^−1^ (Feher et al., 1996). Although different CVs were used in these two works (see Section 2.4.2), the effect of the force field cannot be ruled out. Indeed, the two force fields differ only in a few dihedral potential terms (Best et al., 2012) that control the rigidity of secondary structures, and in particular two helices of T4 which control benzene’s access to the binding pocket. Finally, we mention here the work of (Vitalini et al., 2015), where it was shown that slow relaxation timescales of two small peptides using five protein force fields (AMBER99SB-ILDN (Lindorff-Larsen et al., 2010), AMBERff03 (Duan et al., 2003), OPLS-AA/L (Kaminski et al., 2001), CHARMM27 (MacKerell et al., 2000), and GROMOS43a1 (Daura et al., 1998)) differ up to two orders of magnitude. Given the importance of slow protein conformational changes in unbinding kinetics (Plattner and Noé, 2015), this result further highlights the role of force fields for accurate rate calculations.

### 5.2 Polarization and Charge Transfer Effects

Traditional force fields describe electrostatics using fixed point charges. This representation is extremely efficient and works remarkably well, even in the case of systems with high electric fields (Mironenko et al., 2021). However, such a scheme cannot adapt to changes in the electrostatic environment observed during ligand unbinding. Recently, some of us (Capelli et al., 2020) found that electrostatic effects contribute significantly to the force field misrepresentation of protein-ligand interactions at the transition state of the M2-iperoxo complex. Furthermore, the work of Haldar and coworkers (Haldar et al., 2018) showed that accounting for changes in charge distribution resulted in free energy corrections ranging from 1.9 to 4.7 kcal/mol as the ligand progressed from the hydrophobic binding pocket to the solvated state. Metalloenzymes (representing 40%–50% of all proteins in the PDB database (Chen et al., 2019)) and highly charged protein-ligand systems are also quite challenging to describe with traditional force fields (Li and Merz, 2017). Indeed, for the latter systems, FF-based binding free energy calculations resulted in significant systematic errors (Rocklin et al., 2013). Overall, these results show that going beyond standard fixed-charged models is in many cases desirable to improve accuracy.

## 6 Perspectives: From Polarizable Force Fields to QM/MM Calculations Towards the Exascale

Force fields have been overwhelmingly successful in predicting equilibrium properties such as free energies of binding (Karplus and McCammon, 2002; Wang et al., 2015; Robustelli et al., 2022). Indeed, force fields are traditionally fitted to reproduce equilibrium experimental measurements (ensemble averages) and geometries obtained with quantum mechanical methods. As a result, their performance is expected to peak in the regions near the free energy minima (e.g., the bound state) rather than near the kinetically relevant transition states, where small errors are exponentially amplified in *k*_off_ predictions.^6^ After observing discrepancies of two orders of magnitude in the kinetic predictions of several force fields, Vitalini and coworkers (Vitalini et al., 2015) suggested that kinetic information should be included in the fitting process. In general, designing new parametrization strategies for force fields is still a very active area of research (He et al., 2020; Giannos et al., 2021; Qiu et al., 2021). This is not surprising, given the issues discussed in Section 5. For example, methods to include polarization effects within a fixed-charge scheme (Kelly and Smith, 2020) and multisite models for transition metal ions have been developed (Li and Merz, 2017).

A different direction pursued by the modeling community is instead to use potential energy functions that go beyond the biomolecular force fields’ simple representation of electrostatics (e.g., polarizable force fields, hybrid quantum mechanics/molecular mechanics (QM/MM) calculations, machine learning potentials). Without any claim of being comprehensive, here we provide a brief perspective on the role of these methods in the upcoming era of exascale computing.

### 6.1 Polarizable Force Fields

Polarizable force fields for biomolecules (Jing et al., 2019) including AMBER ff02pol (Wang Z. X. et al., 2006), AMOEBA (Ponder et al., 2010) CHARMM Drude (Baker et al., 2010), CHARMM-FQ (Patel and Brooks, 2003; Patel and Brooks, 2004), SIBFA (Piquemal et al., 2007), and ABEEMσπ (Liu et al., 2017) aim at providing an empirical description electronic polarizability. Simulations based on these potentials could dramatically improve the modeling of transition states in cases where electronic polarization and charge transfer may be linked to non-trivial rearrangements of hydrogen bonds and hydrophobic interactions (Schmidtke et al., 2011; Schiebel et al., 2018). Although polarizable force fields have recently shown excellent accuracy in prospective predictions of binding affinities in model systems (Amezcua et al., 2022), to the best of our knowledge, they have not been used for protein-ligand *k*_off_ predictions yet. Notably, in a very recent paper (Yue et al., 2022), it was shown how using a polarizable force field improved the accuracy of the predictions of anion permeation rates in fluoride channels compared to predictions based on standard fixed charge schemes, highlighting the necessity of using polarizable models for treating such processes. Although this is not a ligand/protein system, this work further showcases the limitations of conventional force fields in treating electrostatic interactions as well as the potential of polarizable models.

### 6.2 QM/MM Simulations

DFT-based QM/MM simulations treat a small region of interest (in our case this could be a ligand and the protein residues interacting with it) at the DFT level, while the overall computational cost is balanced by MM treatment of other regions (Kulik, 2018). The form of the potential energy is a hybrid model between classical mechanics and quantum chemistry:

$$U=\;U_{QM}+\;U_{MM}\;+U_{QM/MM}$$ (3)

where $U_{QM/MM}$ denotes the interaction between atom groups assigned to the QM region and MM region. DFT-based QM/MM simulations include both electronic polarizability and charge transfer effects (Blumberger, 2008; Capelli et al., 2020), and they address the problem of transferability, as they do not rely on optimizations against predefined training data sets. These approaches can tackle important biomedicine problems such as the study of transition-metal-based drugs binding to proteins (Calandrini et al., 2015) or the description of enzymatic reactions. (Carloni et al., 2002; Liao and Thiel, 2013; Roston et al., 2016; Caldararu et al., 2018; Kulik, 2018; Piniello et al., 2021) However, these simulations are orders of magnitude more expensive than any of the potentials described so far, and hence achieving high statistical accuracy with such an approach is obviously extremely challenging.

#### 6.2.1 Parallel Computing in DFT-Based QM/MM

Modern supercomputers are currently breaching the exascale limit in the United States (Schneider, 2022), Japan, and China.^7^ Exascale calculations however remain one of the major challenges in molecular simulations (Hospital et al., 2015; Páll et al., 2015). Recent advances in massively scalable QM/MM codes, such as that developed in Juelich in collaboration with European universities (Olsen et al., 2019; Bolnykh et al., 2020a) (see Supplementary Material S7) and their successful applications to predict free energy landscapes associated with biological processes (Bolnykh et al., 2020a; Chiariello et al., 2020) brings us to suggest that in a not-too-far future QM/MM calculations may exploit the unprecedented power of exascale computing for direct MD simulations of ligand (un)binding (Bolnykh et al., 2020b, Bolnykh et al., 2021).

#### 6.2.2 Machine Learning in QM/MM

Neural network models of the potential energy function have emerged as a promising route to obtaining near-DFT accuracies (Unke et al., 2021; Kocer et al., 2022) at a computational cost only 1–2 orders of magnitude slower than force fields. Applications to the kinetics of chemical reactions have been published (Stocker et al., 2020; Yang et al., 2022) and in principle, they could be used to model DFT-based QM/MM predictions of ligand poses during the unbinding process. However, ML potentials are currently still limited to small molecule applications and robust solutions to model long-range interactions have yet to emerge (Yue et al., 2021). The advent of exascale computing could dramatically expand the domain of applicability of such approaches (Lu et al., 2021). Moreover, several approaches to solve these issues have been proposed based on hybrid machine learning/molecular mechanics models (Shen and Yang, 2018; Rufa et al., 2020; Böselt et al., 2021; Gastegger et al., 2021) (see Supplementary Material S8 for details).

## 7 Conclusion

We have reviewed an array of rather diverse methods able to predict unbinding kinetics constants using atomistic representations of the biomolecules involved. These techniques have shown tremendous progress in the last years: considering trypsin-benzamidine as a benchmark system (as seen in Table 1), we start from 2–3 orders of magnitude in *k*_off_ error in the pioneering MSMs of De Fabritiis and co-workers (Buch et al., 2011) to an error of less than 1 order of magnitude in some of the most recent calculations (Plattner and Noé, 2015; Votapka et al., 2017; Brotzakis et al., 2019; Wolf et al., 2020). Despite these impressive methodological advances, the domain of applicability and accuracy appears to be still limited by current force fields. Better parametrization and polarizable force fields (Lin and MacKerell, 2019) promise to improve the quality of the potential energy model at a reasonable cost at a reasonable computational cost (Lemkul et al., 2016). Another possibility is the use of massively parallel DFT-QM/MM complemented by ML techniques, which include electronic polarizability as well as charge transfer. This approach could address the issue of transferability of current biomolecular force fields. However, the accuracy of these approaches is yet to be established.

Traditionally, computational drug discovery has used a combination of methods such as docking (Ferreira et al., 2015), quantitative structure-activity relationship (QSAR) modeling (Dossetter et al., 2013), free-energy methods (Cournia et al., 2017), and (recently) ML-based approaches (Zhao et al., 2020) to improve the binding affinity of a compound during lead optimization. Computer-aided ligand design campaigns could enormously profit from the design of so-called transition state analogues which, in the case of enzyme inhibitors, have been correlated with release rates that are orders of magnitude slower than product release (Schramm, 2013; Schramm 2015; Svensson et al., 2015). We hope that approaches beyond the use of standard force fields, such as those discussed here, will lead in a not-too-distant future to the accurate description of the energetics and structural determinants of the unbinding transition states, giving an unprecedented boost to the discovery of promising new small molecules and the optimization of known drugs.

## Footnotes

1 These techniques include, among others, scaled MD (Sinko et al., 2013; Bernetti et al., 2018), steered MD (Paci and Karplus, 2000; Potterton et al., 2019; Spiriti and Wong, 2021), targeted MD (Schlitter et al., 1994; Wolf et al., 2019), GAMBES (Debnath and Parrinello, 2020) path-reweighting methods (Chodera et al., 2011; Donati et al., 2017; Kieninger and Keller, 2021) metadynamics of paths (Mandelli et al., 2020) and many transition path sampling-derived methods (Pratt, 1986; Dellago et al., 1998; Van Erp et al., 2003). A brief review of some of these methods (namely scaled MD, targeted MD and GAMBES) is given in the supplementary material.

2 A Kramers’ rate theory correction has been developed for scaled MD as well but it has not been applied to the calculation of full dissociation rates (see Supplementary Material S3.3).

4 i.e., the probability of observing the system in a state y after the lag time given that it was in state *x* does not depend on the states of the system before *x.*

5 All the other studies in Table 1 are instead retrospective, and large-scale benchmarks in prospective settings (which are now common for binding affinity calculations) (Parks et al., 2020; Schindler et al., 2020) are missing.

6 The *k*_off_ values depend exponentially on the height of the dissociation free energy barrier, so even small inaccuracies in the potential energy may impact dramatically kinetics calculations.

7 According to the Top500 list (https://www.top500.org/), which ranks computers based on their performance on the HPLinpack benchmark (Dongarra et al., 2003), currently there are no machines that have exceeded the exascale limit. The Fugaku supercomputer in Japan showed performances above one EFlop/s, but on a different benchmark (Kudo et al., 2020). In China, one or two exascale supercomputers might be already operating(Ma et al., 2022; Schneider, 2022).

## References

[B1] Ahn S.-H. Grate J. W. Darve E. F. (2017). Efficiently Sampling Conformations and Pathways Using the Concurrent Adaptive Sampling (CAS) Algorithm. J. Chem. Phys. 147, 074115. 10.1063/1.4999097 28830168

[B2] Ahn S.-H. Jagger B. R. Amaro R. E. (2020). Ranking of Ligand Binding Kinetics Using a Weighted Ensemble Approach and Comparison with a Multiscale Milestoning Approach. J. Chem. Inf. Model. 60, 5340–5352. 10.1021/acs.jcim.9b00968 32315175

[B3] Amaral M. Kokh D. B. Bomke J. Wegener A. Buchstaller H. P. Eggenweiler H. M. (2017). Protein Conformational Flexibility Modulates Kinetics and Thermodynamics of Drug Binding. Nat. Commun. 8, 2276. 10.1038/s41467-017-02258-w 29273709 PMC5741624

[B4] Amezcua M. Setiadi J. Ge Y. Mobley D. L. (2022). An Overview of the SAMPL8 Host-Guest Binding Challenge. ChemRxiv, 1–42. 10.26434/chemrxiv-2022-lwd0h PMC9596595 36229622

[B5] Ansari N. Rizzi V. Parrinello M. (2022). Water Regulates the Residence Time of Benzamidine in Trypsin. *ArXiv*, 1–16. Available at: http://arxiv.org/abs/2204.05572 . (Accessed April 16, 2022). 10.1038/s41467-022-33104-3 PMC9481606 36114175

[B6] Baker C. M. Lopes P. E. M. Zhu X. Roux B. MacKerell A. D. (2010). Accurate Calculation of Hydration Free Energies Using Pair-specific Lennard-Jones Parameters in the CHARMM Drude Polarizable Force Field. J. Chem. Theory Comput. 6, 1181–1198. 10.1021/ct9005773 20401166 PMC2853947

[B7] Barducci A. Bussi G. Parrinello M. (2008). Well-tempered Metadynamics: A Smoothly Converging and Tunable Free-Energy Method. Phys. Rev. Lett. 100, 1–4. 10.1103/PhysRevLett.100.020603 18232845

[B8] Bartels C. Karplus M. (1997). Multidimensional Adaptive Umbrella Sampling: Applications to Main Chain and Side Chain Peptide Conformations. J. Comput. Chem. 18, 1450–1462. 10.1002/(sici)1096-987x(199709)18:12<1450::aid-jcc3>3.0.co;2-i

[B9] Bernetti M. Rosini E. Mollica L. Masetti M. Pollegioni L. Recanatini M. (2018). Binding Residence Time through Scaled Molecular Dynamics: A Prospective Application to hDAAO Inhibitors. J. Chem. Inf. Model. 58, 2255–2265. 10.1021/acs.jcim.8b00518 30339750

[B10] Best R. B. Hummer G. (2009). Optimized Molecular Dynamics Force Fields Applied to the Helix−Coil Transition of Polypeptides. J. Phys. Chem. B 113, 9004–9015. 10.1021/jp901540t 19514729 PMC3115786

[B11] Best R. B. Zhu X. Shim J. Lopes P. E. M. Mittal J. Feig M. (2012). Optimization of the Additive CHARMM All-Atom Protein Force Field Targeting Improved Sampling of the Backbone ϕ, ψ and Side-Chain χ1 and χ2 Dihedral Angles. J. Chem. Theory Comput. 8, 3257–3273. 10.1021/ct300400x 23341755 PMC3549273

[B12] Blumberger J. (2008). Free Energies for Biological Electron Transfer from QM/MM Calculation: Method, Application and Critical Assessment. Phys. Chem. Chem. Phys. 10, 5651–5667. 10.1039/B807444E 18956100

[B13] Bolnykh V. Olsen J. M. H. Meloni S. Bircher M. P. Ippoliti E. Carloni P. (2020a). MiMiC: Multiscale Modeling in Computational Chemistry. Front. Mol. Biosci. 7, 1–4. 10.3389/fmolb.2020.00045 32266290 PMC7100372

[B14] Bolnykh V. Rossetti G. Rothlisberger U. Carloni P. (2021). Expanding the Boundaries of Ligand–Target Modeling by Exascale Calculations. Wiley Interdiscip. Rev. Comput. Mol. Sci. 11. 10.1002/wcms.1535

[B15] Bolnykh V. Rothlisberger U. Carloni P. (2020b). Biomolecular Simulation: A Perspective from High Performance Computing. Isr. J. Chem. 60, 694–704. 10.1002/ijch.202000022

[B16] Böselt L. Thürlemann M. Riniker S. (2021). Machine Learning in QM/MM Molecular Dynamics Simulations of Condensed-phase Systems. J. Chem. Theory Comput. 17, 2641–2658. 10.1021/acs.jctc.0c01112 33818085

[B17] Bowman G. R. Ensign D. L. Pande V. S. (2010). Enhanced Modeling via Network Theory: Adaptive Sampling of Markov State Models. J. Chem. Theory Comput. 6, 787–794. 10.1021/ct900620b 23626502 PMC3637129

[B18] Branduardi D. Gervasio F. L. Parrinello M. (2007). From A to B in Free Energy Space. J. Chem. Phys. 126, 054103. 10.1063/1.2432340 17302470

[B19] Brotzakis Z. F. Limongelli V. Parrinello M. (2019). Accelerating the Calculation of Protein-Ligand Binding Free Energy and Residence Times Using Dynamically Optimized Collective Variables. J. Chem. Theory Comput. 15, 743–750. 10.1021/acs.jctc.8b00934 30537822

[B20] Bruce N. J. Ganotra G. K. Kokh D. B. Sadiq S. K. Wade R. C. (2018). New Approaches for Computing Ligand-Receptor Binding Kinetics. Curr. Opin. Struct. Biol. 49, 1–10. 10.1016/j.sbi.2017.10.001 29132080

[B21] Buch I. Giorgino T. De Fabritiis G. (2011). Complete Reconstruction of an Enzyme-Inhibitor Binding Process by Molecular Dynamics Simulations. Proc. Natl. Acad. Sci. U.S.A. 108, 10184–10189. 10.1073/pnas.1103547108 21646537 PMC3121846

[B22] Bussi G. Laio A. (2020). Using Metadynamics to Explore Complex Free-Energy Landscapes. Nat. Rev. Phys. 2, 200–212. 10.1038/s42254-020-0153-0

[B23] Calandrini V. Rossetti G. Arnesano F. Natile G. Carloni P. (2015). Computational Metallomics of the Anticancer Drug Cisplatin. J. Inorg. Biochem. 153, 231–238. 10.1016/j.jinorgbio.2015.10.001 26490711

[B24] Caldararu O. Feldt M. Cioloboc D. Van Severen M.-C. Starke K. Mata R. A. (2018). QM/MM Study of the Reaction Mechanism of Sulfite Oxidase. Sci. Rep. 8, 1–15. 10.1038/s41598-018-22751-6 29549261 PMC5856855

[B25] Capelli R. Lyu W. Bolnykh V. Meloni S. Olsen J. M. H. Rothlisberger U. (2020). Accuracy of Molecular Simulation-Based Predictions of Koff Values: A Metadynamics Study. J. Phys. Chem. Lett. 11, 6373–6381. 10.1021/acs.jpclett.0c00999 32672983

[B26] Carloni P. Rothlisberger U. Parrinello M. (2002). The Role and Perspective of Ab Initio Molecular Dynamics in the Study of Biological Systems. Acc. Chem. Res. 35, 455–464. 10.1021/ar010018u 12069631

[B27] Casasnovas R. Limongelli V. Tiwary P. Carloni P. Parrinello M. (2017). Unbinding Kinetics of a P38 MAP Kinase Type II Inhibitor from Metadynamics Simulations. J. Am. Chem. Soc. 139, 4780–4788. 10.1021/jacs.6b12950 28290199

[B28] Cérou F. Guyader A. (2007). Adaptive Multilevel Splitting for Rare Event Analysis. Stoch. Analysis Appl. 25, 417–443. 10.1080/07362990601139628

[B29] Cérou F. Guyader A. Lelièvre T. Pommier D. (2011). A Multiple Replica Approach to Simulate Reactive Trajectories. J. Chem. Phys. 134, 054108. 10.1063/1.3518708 21303093

[B30] Cézard C. Trivelli X. Aubry F. Djedaïni-Pilard F. Dupradeau F.-Y. (2011). Molecular Dynamics Studies of Native and Substituted Cyclodextrins in Different Media: 1. Charge Derivation and Force Field Performances. Phys. Chem. Chem. Phys. 13, 15103–15121. 10.1039/C1CP20854C 21792425

[B31] Chen A. Y. Adamek R. N. Dick B. L. Credille C. V. Morrison C. N. Cohen S. M. (2019). Targeting Metalloenzymes for Therapeutic Intervention. Chem. Rev. 119, 1323–1455. 10.1021/acs.chemrev.8b00201 30192523 PMC6405328

[B32] Chiariello M. G. Bolnykh V. Ippoliti E. Meloni S. Olsen J. M. H. Beck T. (2020). Molecular Basis of CLC Antiporter Inhibition by Fluoride. J. Am. Chem. Soc. 142, 7254–7258. 10.1021/jacs.9b13588 32233472

[B33] Cho S. S. Levy Y. Wolynes P. G. (2006). P versus Q : Structural Reaction Coordinates Capture Protein Folding on Smooth Landscapes. Proc. Natl. Acad. Sci. U.S.A. 103, 586–591. 10.1073/pnas.0509768103 16407126 PMC1334664

[B34] Chodera J. D. Swope W. C. Noé F. Prinz J.-H. Shirts M. R. Pande V. S. (2011). Dynamical Reweighting: Improved Estimates of Dynamical Properties from Simulations at Multiple Temperatures. J. Chem. Phys. 134, 244107. 10.1063/1.3592152 21721612 PMC3143679

[B35] Chong L. T. Saglam A. S. Zuckerman D. M. (2017). Path-sampling Strategies for Simulating Rare Events in Biomolecular Systems. Curr. Opin. Struct. Biol. 43, 88–94. 10.1016/j.sbi.2016.11.019 27984811 PMC5420491

[B36] Copeland R. A. (2021). Evolution of the Drug-Target Residence Time Model. Expert Opin. Drug Discov. 16, 1441–1451. 10.1080/17460441.2021.1948997 34210223

[B37] Copeland R. A. Pompliano D. L. Meek T. D. (2006). Drug-target Residence Time and its Implications for Lead Optimization. Nat. Rev. Drug Discov. 5, 730–739. 10.1038/nrd2082 16888652

[B38] Cournia Z. Allen B. Sherman W. (2017). Relative Binding Free Energy Calculations in Drug Discovery: Recent Advances and Practical Considerations. J. Chem. Inf. Model. 57, 2911–2937. 10.1021/acs.jcim.7b00564 29243483

[B39] Crommelin D. Vanden-Eijnden E. (2009). Data-Based Inference of Generators for Markov Jump Processes Using Convex Optimization. Multiscale Model. Simul. 7, 1751–1778. 10.1137/080735977

[B40] Dama J. F. Parrinello M. Voth G. A. (2014). Well-Tempered Metadynamics Converges Asymptotically. Phys. Rev. Lett. 112, 240602. 10.1103/PhysRevLett.112.240602 24996077

[B41] Daura X. Mark A. E. Van Gunsteren W. F. (1998). Parametrization of Aliphatic CHn United Atoms of GROMOS96 Force Field. J. Comput. Chem. 19, 535–547. 10.1002/(sici)1096-987x(19980415)19:5<535::aid-jcc6>3.0.co;2-n

[B42] De Vivo M. Masetti M. Bottegoni G. Cavalli A. (2016). Role of Molecular Dynamics and Related Methods in Drug Discovery. J. Med. Chem. 59, 4035–4061. 10.1021/acs.jmedchem.5b01684 26807648

[B43] de Witte W. E. A. Danhof M. van der Graaf P. H. de Lange E. C. M. (2018). The Implications of Target Saturation for the Use of Drug-Target Residence Time. Nat. Rev. Drug Discov. 18, 84. 10.1038/nrd.2018.234 30591716

[B44] Debnath J. Parrinello M. (2020). Gaussian Mixture-Based Enhanced Sampling for Statics and Dynamics. J. Phys. Chem. Lett. 11, 5076–5080. 10.1021/acs.jpclett.0c01125 32510225

[B45] Dellago C. Bolhuis P. G. Csajka F. S. Chandler D. (1998). Transition Path Sampling and the Calculation of Rate Constants. J. Chem. Phys. 108, 1964–1977. 10.1063/1.475562

[B46] Dickson A. Brooks C. L. (2013). Native States of Fast-Folding Proteins Are Kinetic Traps. J. Am. Chem. Soc. 135, 4729–4734. 10.1021/ja311077u 23458553 PMC3619186

[B47] Dickson A. Brooks C. L. (2014). WExplore: Hierarchical Exploration of High-Dimensional Spaces Using the Weighted Ensemble Algorithm. J. Phys. Chem. B 118, 3532–3542. 10.1021/jp411479c.WExplore 24490961 PMC4404516

[B48] Dickson A. Lotz S. D. (2017). Multiple Ligand Unbinding Pathways and Ligand-Induced Destabilization Revealed by WExplore. Biophysical J. 112, 620–629. 10.1016/j.bpj.2017.01.006 PMC5340210 28256222

[B49] Dixon T. Uyar A. Ferguson-Miller S. Dickson A. (2021). Membrane-Mediated Ligand Unbinding of the PK-11195 Ligand from TSPO. Biophysical J. 120, 158–167. 10.1016/j.bpj.2020.11.015 PMC7820730 33221248

[B50] Donati L. Hartmann C. Keller B. G. (2017). Girsanov Reweighting for Path Ensembles and Markov State Models. J. Chem. Phys. 146, 244112. 10.1063/1.4989474 28668056

[B51] Dongarra J. J. Luszczek P. Petitet A. (2003). The LINPACK Benchmark: Past, Present and Future. Concurr. Comput. Pract. Exper. 15, 803–820. 10.1002/cpe.728

[B52] Donyapour N. Roussey N. M. Dickson A. (2019). REVO: Resampling of Ensembles by Variation Optimization. J. Chem. Phys. 150, 244112–12. 10.1063/1.5100521 31255090 PMC7043833

[B53] Dossetter A. G. Griffen E. J. Leach A. G. (2013). Matched Molecular Pair Analysis in Drug Discovery. Drug Discov. Today 18, 724–731. 10.1016/j.drudis.2013.03.003 23557664

[B54] Duan Y. Wu C. Chowdhury S. Lee M. C. Xiong G. Zhang W. (2003). A Point-Charge Force Field for Molecular Mechanics Simulations of Proteins Based on Condensed-phase Quantum Mechanical Calculations. J. Comput. Chem. 24, 1999–2012. 10.1002/jcc.10349 14531054

[B55] Durrant J. D. McCammon J. A. (2011). Molecular Dynamics Simulations and Drug Discovery. BMC Biol. 9, 71. 10.1016/B978-0-12-809633-8.20154-410.1186/1741-7007-9-71 22035460 PMC3203851

[B56] Elber R. (2007). A Milestoning Study of the Kinetics of an Allosteric Transition: Atomically Detailed Simulations of Deoxy Scapharca Hemoglobin. Biophysical J. 92, L85–L87. 10.1529/biophysj.106.101899 PMC1852362 17325010

[B57] Elber R. (2020). Milestoning: An Efficient Approach for Atomically Detailed Simulations of Kinetics in Biophysics. Annu. Rev. Biophys. 49, 69–85. 10.1146/annurev-biophys-121219-081528 32375019

[B58] Emwas A.-H. Szczepski K. Poulson B. G. Chandra K. Mckay R. T. Dhahri M. (2020). NMR as a "Gold Standard" Method in Drug Design and Discovery. Molecules 25, 4597. 10.3390/molecules25204597 PMC7594251 33050240

[B59] Feher V. A. Baldwin E. P. Dahlquist F. W. (1996). Access of Ligands to Cavities within the Core of a Protein Is Rapid. Nat. Struct. Mol. Biol. 3, 516–521. 10.1038/nsb0696-516 8646537

[B60] Ferreira L. Dos Santos R. Oliva G. Andricopulo A. (2015). Molecular Docking and Structure-Based Drug Design Strategies. Molecules 20, 13384–13421. 10.3390/molecules200713384 26205061 PMC6332083

[B61] Folmer R. H. A. (2018). Drug Target Residence Time: a Misleading Concept. Drug Discov. Today 23, 12–16. 10.1016/j.drudis.2017.07.016 28782685

[B62] Gastegger M. Schütt K. T. Müller K.-R. (2021). Machine Learning of Solvent Effects on Molecular Spectra and Reactions. Chem. Sci. 12, 11473–11483. 10.1039/d1sc02742e 34567501 PMC8409491

[B63] Gelpi J. Hospital A. Goñi R. Orozco M. (2015). Molecular Dynamics Simulations: Advances and Applications. Aabc 8, 37–47. 10.2147/AABC.S70333 26604800 PMC4655909

[B64] Giannos T. Lešnik S. Bren U. Hodošček M. Domratcheva T. Bondar A.-N. (2021). CHARMM Force-Field Parameters for Morphine, Heroin, and Oliceridine, and Conformational Dynamics of Opioid Drugs. J. Chem. Inf. Model. 61, 3964–3977. 10.1021/acs.jcim.1c00667 34351148

[B65] Grubmüller H. (1995). Predicting Slow Structural Transitions in Macromolecular Systems: Conformational Flooding. Phys. Rev. E 52, 2893–2906. 10.1103/physreve.52.2893 9963736

[B66] Guillian F. Thusias D. (1970). The Use of Proflavin as an Indicator in Temperature-Jump Studies of the Binding of a Competitive Inhibitor to Trypsin. J. Am. Chem. Soc. 92 (18), 5534–5536. 5449454 10.1021/ja00721a051

[B67] Guo D. Mulder-Krieger T. IJzerman A. P. Heitman L. H. (2012). Functional Efficacy of Adenosine A2A Receptor Agonists Is Positively Correlated to Their Receptor Residence Time. Br. J. Pharmacol. 166, 1846–1859. 10.1111/j.1476-5381.2012.01897.x 22324512 PMC3402809

[B68] Haldar S. Comitani F. Saladino G. Woods C. Van Der Kamp M. W. Mulholland A. J. (2018). A Multiscale Simulation Approach to Modeling Drug-Protein Binding Kinetics. J. Chem. Theory Comput. 14, 6093–6101. 10.1021/acs.jctc.8b00687 30208708

[B69] Hänggi P. Talkner P. Borkovec M. (1990). Reaction-rate Theory: Fifty Years after Kramers. Rev. Mod. Phys. 62, 251–341. 10.1103/RevModPhys.62.251

[B70] He X. Man V. H. Yang W. Lee T.-S. Wang J. (2020). A Fast and High-Quality Charge Model for the Next Generation General AMBER Force Field. J. Chem. Phys. 153, 114502. 10.1063/5.0019056 32962378 PMC7728379

[B71] Hooft R. W. W. Van Eijck B. P. Kroon J. (1992). An Adaptive Umbrella Sampling Procedure in Conformational Analysis Using Molecular Dynamics and its Application to Glycol. J. Chem. Phys. 97, 6690–6694. 10.1063/1.463947

[B72] Hornak V. Abel R. Okur A. Strockbine B. Roitberg A. Simmerling C. (2006). Comparison of Multiple Amber Force Fields and Development of Improved Protein Backbone Parameters. Proteins 65, 712–725. 10.1002/prot.21123 16981200 PMC4805110

[B73] Huber G. A. Kim S. (1996). Weighted-ensemble Brownian Dynamics Simulations for Protein Association Reactions. Biophysical J. 70, 97–110. 10.1016/S0006-3495(96)79552-8 PMC1224912 8770190

[B74] Huber T. Torda A. E. van Gunsteren W. F. (1994). Local Elevation: A Method for Improving the Searching Properties of Molecular Dynamics Simulation. J. Computer-Aided Mol. Des. 8, 695–708. 10.1007/BF00124016 7738605

[B75] Husic B. E. Pande V. S. (2018). Markov State Models: From an Art to a Science. J. Am. Chem. Soc. 140, 2386–2396. 10.1021/jacs.7b12191 29323881

[B76] Invernizzi M. Parrinello M. (2020). Rethinking Metadynamics: From Bias Potentials to Probability Distributions. J. Phys. Chem. Lett. 11, 2731–2736. 10.1021/acs.jpclett.0c00497 32191470

[B77] Jagger B. Ojha A. A. Amaro R. (2020). Predicting Ligand Binding Kinetics Using a Markovian Milestoning with Voronoi Tessellations Multiscale Approach. ChemRxiv 16 (8), 5348–5357. 10.26434/chemrxiv.12275165.v1 32579371

[B78] Jing Z. Liu C. Cheng S. Y. Qi R. Walker B. D. Piquemal J.-P. (2019). Polarizable Force Fields for Biomolecular Simulations: Recent Advances and Applications. Annu. Rev. Biophys. 48, 371–394. 10.1146/annurev-biophys-070317-033349 30916997 PMC6520134

[B79] Juraszek J. Saladino G. van Erp T. S. Gervasio F. L. (2013). Efficient Numerical Reconstruction of Protein Folding Kinetics with Partial Path Sampling and Pathlike Variables. Phys. Rev. Lett. 110, 108106. 10.1103/PhysRevLett.110.108106 23521305

[B80] Kalbfleisch J. D. Lawless J. F. (1985). The Analysis of Panel Data under a Markov Assumption. J. Am. Stat. Assoc. 80, 863–871. 10.1080/01621459.1985.10478195

[B81] Kaminski G. A. Friesner R. A. Tirado-Rives J. Jorgensen W. L. (2001). Evaluation and Reparametrization of the OPLS-AA Force Field for Proteins via Comparison with Accurate Quantum Chemical Calculations on Peptides. J. Phys. Chem. B 105, 6474–6487. 10.1021/jp003919d

[B82] Karplus M. McCammon J. A. (2002). Molecular Dynamics Simulations of Biomolecules. Nat. Struct. Biol. 9, 646–652. 10.1299/jsmemag.116.1131_7810.1038/nsb0902-646 12198485

[B83] Kelly B. D. Smith W. R. (2020). Alchemical Hydration Free-Energy Calculations Using Molecular Dynamics with Explicit Polarization and Induced Polarity Decoupling: An On-The-Fly Polarization Approach. J. Chem. Theory Comput. 16, 1146–1161. 10.1021/acs.jctc.9b01139 31930918

[B84] Kieninger S. Keller B. G. (2021). Path Probability Ratios for Langevin Dynamics-Exact and Approximate. J. Chem. Phys. 154, 094102. 10.1063/5.0038408 33685138

[B85] Kocer E. Ko T. W. Behler J. (2022). Neural Network Potentials: A Concise Overview of Methods. Annu. Rev. Phys. Chem. 73, 163–186. 10.1146/annurev-physchem-082720-034254 34982580

[B86] Kokh D. B. Amaral M. Bomke J. Grädler U. Musil D. Buchstaller H.-P. (2018). Estimation of Drug-Target Residence Times by τ-Random Acceleration Molecular Dynamics Simulations. J. Chem. Theory Comput. 14, 3859–3869. 10.1021/acs.jctc.8b00230 29768913

[B87] Kokh D. B. Doser B. Richter S. Ormersbach F. Cheng X. Wade R. C. (2020). A Workflow for Exploring Ligand Dissociation from a Macromolecule: Efficient Random Acceleration Molecular Dynamics Simulation and Interaction Fingerprint Analysis of Ligand Trajectories. J. Chem. Phys. 153, 125102. 10.1063/5.0019088 33003755

[B88] Kokh D. B. Kaufmann T. Kister B. Wade R. C. (2019). Machine Learning Analysis of τRAMD Trajectories to Decipher Molecular Determinants of Drug-Target Residence Times. Front. Mol. Biosci. 6, 1–17. 10.3389/fmolb.2019.00036 31179286 PMC6543870

[B89] Kudo S. Nitadori K. Ina T. Imamura T. (2020). “Prompt Report on Exa-Scale HPL-AI Benchmark,” in 2020 IEEE International Conference on Cluster Computing (CLUSTER), Kobe, Japan. St. Louis, Missouri, USA, 418–419. 10.1109/CLUSTER49012.2020.00058

[B90] Kulik H. J. (2018). Large-scale QM/MM Free Energy Simulations of Enzyme Catalysis Reveal the Influence of Charge Transfer. Phys. Chem. Chem. Phys. 20, 20650–20660. 10.1039/c8cp03871f 30059109 PMC6085747

[B91] Kumar S. Rosenberg J. M. Bouzida D. Swendsen R. H. Kollman P. A. (1992). THE Weighted Histogram Analysis Method for Free-Energy Calculations on Biomolecules. I. The Method. J. Comput. Chem. 13, 1011–1021. 10.1002/jcc.540130812

[B205] Kwarcinski F. E. Brandvold K. R. Phadke S. Beleh O. M. Johnson T. M. Meagher J. L. (2016). Conformation-Selective Analogues of Dasatinib Reveal Insight into Kinase Inhibitor Binding and Selectivity. ACS Chem. Biol. 11, 1296–1304. 26895387 10.1021/acschembio.5b01018 PMC7306399

[B92] Laio A. Parrinello M. (2002). Escaping Free-Energy Minima. Proc. Natl. Acad. Sci. U. S. A. 99 (20), 12562–12566. 10.1073/pnas.202427399 12271136 PMC130499

[B93] Lee K. S. S. Yang J. Niu J. Ng C. J. Wagner K. M. Dong H. (2019). Drug-Target Residence Time Affects *In Vivo* Target Occupancy through Multiple Pathways. ACS Cent. Sci. 5, 1614–1624. 10.1021/acscentsci.9b00770 31572788 PMC6764161

[B94] Lemkul J. A. Huang J. Roux B. MacKerell A. D. (2016). An Empirical Polarizable Force Field Based on the Classical Drude Oscillator Model: Development History and Recent Applications. Chem. Rev. 116, 4983–5013. 10.1021/acs.chemrev.5b00505 26815602 PMC4865892

[B95] Li H.-J. Lai C.-T. Pan P. Yu W. Liu N. Bommineni G. R. (2014). A Structural and Energetic Model for the Slow-Onset Inhibition of the mycobacterium Tuberculosis ENoyl-ACP Reductase InhA. ACS Chem. Biol. 9, 986–993. 10.1021/cb400896g 24527857 PMC4004265

[B96] Li P. Merz K. M. (2017). Metal Ion Modeling Using Classical Mechanics. Chem. Rev. 117, 1564–1686. 10.1021/acs.chemrev.6b00440 28045509 PMC5312828

[B97] Liao R.-Z. Thiel W. (2013). Convergence in the QM-Only and QM/MM Modeling of Enzymatic Reactions: A Case Study for Acetylene Hydratase. J. Comput. Chem. 34, a–n. 10.1002/jcc.23403 23913757

[B98] Lin F.-Y. MacKerell A. D. (2019). Improved Modeling of Halogenated Ligand-Protein Interactions Using the Drude Polarizable and CHARMM Additive Empirical Force Fields. J. Chem. Inf. Model. 59, 215–228. 10.1021/acs.jcim.8b00616 30418023 PMC6349471

[B99] Lindorff-Larsen K. Piana S. Palmo K. Maragakis P. Klepeis J. L. Dror R. O. (2010). Improved Side-Chain Torsion Potentials for the Amber ff99SB Protein Force Field. Proteins 78, 1950–1958. 10.1002/prot.22711 20408171 PMC2970904

[B100] Liu C. Li Y. Han B.-Y. Gong L.-D. Lu L.-N. Yang Z.-Z. (2017). Development of the ABEEMσπ Polarization Force Field for Base Pairs with Amino Acid Residue Complexes. J. Chem. Theory Comput. 13, 2098–2111. 10.1021/acs.jctc.6b01206 28402659

[B101] Lotz S. D. Dickson A. (2018). Unbiased Molecular Dynamics of 11 Min Timescale Drug Unbinding Reveals Transition State Stabilizing Interactions. J. Am. Chem. Soc. 140, 618–628. 10.1021/jacs.7b08572 29303257

[B102] Lotz S. D. Dickson A. (2020). Wepy: A Flexible Software Framework for Simulating Rare Events with Weighted Ensemble Resampling. ACS Omega 5, 31608–31623. 10.1021/acsomega.0c03892 33344813 PMC7745226

[B103] Lu D. Wang H. Chen M. Lin L. Car R. E W. (2021). 86 PFLOPS Deep Potential Molecular Dynamics Simulation of 100 Million Atoms with Ab Initio Accuracy. Comput. Phys. Commun. 259, 107624. 10.1016/j.cpc.2020.107624

[B104] Lüdemann S. K. Lounnas V. Wade R. C. (2000). How Do Substrates Enter and Products Exit the Buried Active Site of Cytochrome P450cam? 2. Steered Molecular Dynamics and Adiabatic Mapping of Substrate Pathways 1 1Edited by J. Thornton. J. Mol. Biol. 303, 813–830. 10.1006/jmbi.2000.4155 11061977

[B105] Luty B. A. El Amrani S. McCammon J. A. (1993). Simulation of the Bimolecular Reaction between Superoxide and Superoxide Dismutase: Synthesis of the Encounter and Reaction Steps. J. Am. Chem. Soc. 115, 11874–11877. 10.1021/ja00078a027

[B106] Ma Z. He J. Qiu J. Cao H. Wang Y. Sun Z. (2022). “BaGuaLu: Targeting Brain Scale Pretrained Models with over 37 Million Cores,” in ACM SIGPLAN Annual Symposium on Principles and Practice of Parallel Programming.

[B107] MacKerell A. D. Banavali N. Foloppe N. (2000). Development and Current Status of the CHARMM Force Field for Nucleic Acids. Biopolymers 56, 257–265. 10.1002/1097-0282(2000)56:4<257::aid-bip10029>3.0.co;2-w 11754339

[B108] MacKerell A. D. Bashford D. Bellott M. Dunbrack R. L. Evanseck J. D. Field M. J. (1998). All-atom Empirical Potential for Molecular Modeling and Dynamics Studies of Proteins. J. Phys. Chem. B 102, 3586–3616. 10.1021/jp973084f 24889800

[B109] MacKerell A. D. Jr. Feig M. Brooks C. L. III (2004). Extending the Treatment of Backbone Energetics in Protein Force Fields: Limitations of Gas-phase Quantum Mechanics in Reproducing Protein Conformational Distributions in Molecular Dynamics Simulations. J. Comput. Chem. 25, 1400–1415. 10.1002/jcc.20065 15185334

[B110] Maier J. A. Martinez C. Kasavajhala K. Wickstrom L. Hauser K. E. Simmerling C. (2015). ff14SB: Improving the Accuracy of Protein Side Chain and Backbone Parameters from ff99SB. J. Chem. Theory Comput. 11, 3696–3713. 10.1021/acs.jctc.5b00255 26574453 PMC4821407

[B111] Mandelli D. Hirshberg B. Parrinello M. (2020). Metadynamics of Paths. Phys. Rev. Lett. 125, 26001. 10.1103/PhysRevLett.125.026001 32701329

[B112] Mardt A. Pasquali L. Wu H. Noé F. (2018). VAMPnets for Deep Learning of Molecular Kinetics. Nat. Commun. 9, 1–14. 10.1038/s41467-017-02388-1 29295994 PMC5750224

[B113] Maximova E. Postnikov E. B. Lavrova A. I. Farafonov V. Nerukh D. (2021). Protein-Ligand Dissociation Rate Constant from All-Atom Simulation. J. Phys. Chem. Lett. 12, 10631–10636. 10.1021/acs.jpclett.1c02952 34704768

[B114] Mazzorana M. Shotton E. J. Hall D. R. (2020). A Comprehensive Approach to X-Ray Crystallography for Drug Discovery at a Synchrotron Facility - the Example of Diamond Light Source. Drug Discov. Today Technol. 37, 83–92. 10.1016/j.ddtec.2020.10.003 34895658

[B115] Miao Y. (2018). Acceleration of Biomolecular Kinetics in Gaussian Accelerated Molecular Dynamics. J. Chem. Phys. 149, 072308. 10.1063/1.5024217 30134710 PMC6901173

[B116] Miao Y. Bhattarai A. Wang J. (2020). Ligand Gaussian Accelerated Molecular Dynamics (LiGaMD): Characterization of Ligand Binding Thermodynamics and Kinetics. J. Chem. Theory Comput. 16, 5526–5547. 10.1021/acs.jctc.0c00395 32692556 PMC7768792

[B117] Mironenko A. Zachariae U. de Groot B. L. Kopec W. (2021). The Persistent Question of Potassium Channel Permeation Mechanisms. J. Mol. Biol. 433, 167002. 10.1016/j.jmb.2021.167002 33891905

[B118] Mondal J. Ahalawat N. Pandit S. Kay L. E. Vallurupalli P. (2018). Atomic Resolution Mechanism of Ligand Binding to a Solvent Inaccessible Cavity in T4 Lysozyme. PLoS Comput. Biol. 14, e1006180–20. 10.1371/journal.pcbi.1006180 29775455 PMC5979041

[B119] Morando M. A. Saladino G. D’Amelio N. Pucheta-Martinez E. Lovera S. Lelli M. (2016). Conformational Selection and Induced Fit Mechanisms in the Binding of an Anticancer Drug to the C-Src Kinase. Sci. Rep. 6, 1–9. 10.1038/srep24439 27087366 PMC4834493

[B120] Moroni D. Bolhuis P. G. van Erp T. S. (2004). Rate Constants for Diffusive Processes by Partial Path Sampling. J. Chem. Phys. 120, 4055–4065. 10.1063/1.1644537 15268572

[B121] Nunes-Alves A. Kokh D. B. Wade R. C. (2020). Recent Progress in Molecular Simulation Methods for Drug Binding Kinetics. Curr. Opin. Struct. Biol. 64, 126–133. 10.1016/j.sbi.2020.06.022 32771530

[B122] Olsen J. M. H. Bolnykh V. Meloni S. Ippoliti E. Bircher M. P. Carloni P. (2019). MiMiC: A Novel Framework for Multiscale Modeling in Computational Chemistry. J. Chem. Theory Comput. 15, 3810–3823. 10.1021/acs.jctc.9b00093 30998344

[B123] Páll S. Abraham M. J. Kutzner C. Hess B. Lindahl E. (2015). Tackling Exascale Software Challenges in Molecular Dynamics Simulations with GROMACS BT Solving Software Challenges for Exascale. in, eds. Markidis S. Laure E. (Cham: Springer International Publishing), 3–27. 10.1007/978-3-319-15976-8_1

[B124] Paci E. Karplus M. (2000). Unfolding Proteins by External Forces and Temperature: The Importance of Topology and Energetics. Proc. Natl. Acad. Sci. U.S.A. 97, 6521–6526. 10.1073/pnas.100124597 10823892 PMC18644

[B125] Pan A. C. Borhani D. W. Dror R. O. Shaw D. E. (2013). Molecular Determinants of Drug-Receptor Binding Kinetics. Drug Discov. Today 18, 667–673. 10.1016/j.drudis.2013.02.007 23454741

[B126] Pan A. C. Jacobson D. Yatsenko K. Sritharan D. Weinreich T. M. Shaw D. E. (2019). Atomic-level Characterization of Protein-Protein Association. Proc. Natl. Acad. Sci. U.S.A. 116, 4244–4249. 10.1073/pnas.1815431116 30760596 PMC6410769

[B127] Pan A. C. Xu H. Palpant T. Shaw D. E. (2017). Quantitative Characterization of the Binding and Unbinding of Millimolar Drug Fragments with Molecular Dynamics Simulations. J. Chem. Theory Comput. 13, 3372–3377. 10.1021/acs.jctc.7b00172 28582625

[B128] Parks C. D. Gaieb Z. Chiu M. Yang H. Shao C. Walters W. P. (2020). D3R Grand Challenge 4: Blind Prediction of Protein-Ligand Poses, Affinity Rankings, and Relative Binding Free Energies. J. Comput. Aided. Mol. Des. 34, 99–119. 10.1007/s10822-020-00289-y 31974851 PMC7261493

[B129] Patel S. Brooks C. L. 3rd (2004). CHARMM Fluctuating Charge Force Field for Proteins: I Parameterization and Application to Bulk Organic Liquid Simulations. J. Comput. Chem. 25, 1–16. 10.1002/jcc.10355 14634989

[B130] Patel S. Brooks C. L. III (2003). CHARMM Fluctuating Charge Force Field for Proteins: I Parameterization and Application to Bulk Organic Liquid Simulations. J. Comput. Chem. 25, 1–16. 10.1002/jcc.10355 14634989

[B131] Paul F. Wehmeyer C. Abualrous E. T. Wu H. Crabtree M. D. Schöneberg J. (2017). Protein-peptide Association Kinetics beyond the Seconds Timescale from Atomistic Simulations. Nat. Commun. 8, 1–9. 10.1038/s41467-017-01163-6 29062047 PMC5653669

[B132] Piniello B. Lira-Navarrete E. Takeuchi H. Takeuchi M. Haltiwanger R. S. Hurtado-Guerrero R. (2021). Asparagine Tautomerization in Glycosyltransferase Catalysis. The Molecular Mechanism of Protein O-Fucosyltransferase 1. ACS Catal. 11, 9926–9932. 10.1021/acscatal.1c01785 34868727 PMC8631701

[B133] Piquemal J.-P. Chevreau H. Gresh N. (2007). Toward a Separate Reproduction of the Contributions to the Hartree−Fock and DFT Intermolecular Interaction Energies by Polarizable Molecular Mechanics with the SIBFA Potential. J. Chem. Theory Comput. 3, 824–837. 10.1021/ct7000182 26627402

[B134] Plattner N. Noé F. (2015). Protein Conformational Plasticity and Complex Ligand-Binding Kinetics Explored by Atomistic Simulations and Markov Models. Nat. Commun. 6. 10.1038/ncomms8653 PMC4506540 26134632

[B135] Pollard T. D. (2010). A Guide to Simple and Informative Binding Assays. MBoC 21, 4061–4067. 10.1091/mbc.E10-08-0683 21115850 PMC2993736

[B136] Ponder J. W. Wu C. Ren P. Pande V. S. Chodera J. D. Schnieders M. J. (2010). Current Status of the AMOEBA Polarizable Force Field. J. Phys. Chem. B 114, 2549–2564. 10.1021/jp910674d 20136072 PMC2918242

[B137] Potterton A. Husseini F. S. Southey M. W. Y. Bodkin M. J. Heifetz A. Coveney P. V. (2019). Ensemble-Based Steered Molecular Dynamics Predicts Relative Residence Time of A2A Receptor Binders. J. Chem. Theory Comput. 15, 3316–3330. 10.1021/acs.jctc.8b01270 30893556

[B138] Pratt L. R. (1986). A Statistical Method for Identifying Transition States in High Dimensional Problems. J. Chem. Phys. 85, 5045–5048. 10.1063/1.451695

[B139] Proudfoot A. Bussiere D. E. Lingel A. (2017). High-Confidence Protein-Ligand Complex Modeling by NMR-Guided Docking Enables Early Hit Optimization. J. Am. Chem. Soc. 139, 17824–17833. 10.1021/jacs.7b07171 29190085

[B140] Qiu Y. Smith D. G. A. Boothroyd S. Jang H. Hahn D. F. Wagner J. (2021). Development and Benchmarking of Open Force Field v1.0.0-the Parsley Small-Molecule Force Field. J. Chem. Theory Comput. 17, 6262–6280. 10.1021/acs.jctc.1c00571 34551262 PMC8511297

[B141] Ray D. Andricioaei I. (2020). Weighted Ensemble Milestoning (WEM): A Combined Approach for Rare Event Simulations. J. Chem. Phys. 152, 234114. 10.1063/5.0008028 32571033

[B142] Ray D. Stone S. E. Andricioaei I. (2022). Markovian Weighted Ensemble Milestoning (M-WEM): Long-Time Kinetics from Short Trajectories. J. Chem. Theory Comput. 18, 79–95. 10.1021/acs.jctc.1c00803 34910499

[B143] Regan J. Pargellis C. A. Cirillo P. F. Gilmore T. Hickey E. R. Peet G. W. (2003). The Kinetics of Binding to p38MAP Kinase by Analogues of BIRB 796. Bioorg. Med. Chem. Lett. 13, 3101–3104. 10.1016/S0960-894X(03)00656-5 12941343

[B144] Robustelli P. Ibanez-de-Opakua A. Campbell-Bezat C. Giordanetto F. Becker S. Zweckstetter M. (2022). Molecular Basis of Small-Molecule Binding to α-Synuclein. J. Am. Chem. Soc. 144, 2501–2510. 10.1021/jacs.1c07591 35130691 PMC8855421

[B145] Rocklin G. J. Boyce S. E. Fischer M. Fish I. Mobley D. L. Shoichet B. K. (2013). Blind Prediction of Charged Ligand Binding Affinities in a Model Binding Site. J. Mol. Biol. 425, 4569–4583. 10.1016/j.jmb.2013.07.030 23896298 PMC3962782

[B146] Roston D. Demapan D. Cui Q. (2016). Leaving Group Ability Observably Affects Transition State Structure in a Single Enzyme Active Site. J. Am. Chem. Soc. 138, 7386–7394. 10.1021/jacs.6b03156 27186960 PMC5705186

[B147] Rufa D. A. Bruce Macdonald H. E. Fass J. Wieder M. Grinaway P. B. Roitberg A. E. (2020). Towards Chemical Accuracy for Alchemical Free Energy Calculations with Hybrid Physics-Based Machine Learning/Molecular Mechanics Potentials. bioRxiv, 1–21. 10.1101/2020.07.29.227959

[B148] Salvalaglio M. Tiwary P. Parrinello M. (2014). Assessing the Reliability of the Dynamics Reconstructed from Metadynamics. J. Chem. Theory Comput. 10, 1420–1425. 10.1021/ct500040r 26580360

[B149] Schäfer T. M. Settanni G. (2020). Data Reweighting in Metadynamics Simulations. J. Chem. Theory Comput. 16, 2042–2052. 10.1021/acs.jctc.9b00867 32192340

[B150] Schiebel J. Gaspari R. Wulsdorf T. Ngo K. Sohn C. Schrader T. E. (2018). Intriguing Role of Water in Protein-Ligand Binding Studied by Neutron Crystallography on Trypsin Complexes. Nat. Commun. 9, 3559. 10.1038/s41467-018-05769-2 30177695 PMC6120877

[B151] Schindler C. E. M. Baumann H. Blum A. Böse D. Buchstaller H.-P. Burgdorf L. (2020). Large-Scale Assessment of Binding Free Energy Calculations in Active Drug Discovery Projects. J. Chem. Inf. Model. 60, 5457–5474. 10.1021/acs.jcim.0c00900 32813975

[B152] Schlitter J. Engels M. Krüger P. (1994). Targeted Molecular Dynamics: A New Approach for Searching Pathways of Conformational Transitions. J. Mol. Graph. 12, 84–89. 10.1016/0263-7855(94)80072-3 7918256

[B153] Schmidtke P. Luque F. J. Murray J. B. Barril X. (2011). Shielded Hydrogen Bonds as Structural Determinants of Binding Kinetics: Application in Drug Design. J. Am. Chem. Soc. 133, 18903–18910. 10.1021/ja207494u 21981450

[B154] Schneider D. (2022). The Exascale Era Is upon Us: The Frontier Supercomputer May Be the First to Reach 1,000,000,000,000,000,000 Operations Per Second. IEEE Spectr. 59, 34–35. 10.1109/MSPEC.2022.9676353

[B155] Schramm V. L. (2015). Transition States and Transition State Analogue Interactions with Enzymes. Acc. Chem. Res. 48, 1032–1039. 10.1021/acs.accounts.5b00002 25848811 PMC4482137

[B156] Schramm V. L. (2013). Transition States, Analogues, and Drug Development. ACS Chem. Biol. 8, 71–81. 10.1021/cb300631k 23259601 PMC3560411

[B206] Shan Y. Seeliger M. A. Eastwood M. P. Frank F. Xu H. Jensen M. (2009). A Conserved Protonation-Dependent Switch Controls Drug Binding in the Abl Kinase. Proc. Natl. Acad. Sci. 106, 139–144. 10.1073/pnas.0811223106 19109437 PMC2610013

[B157] Shaw D. E. Adams P. J. Azaria A. Bank J. A. Batson B. Bell A. (2021). “Anton 3: Twenty Microseconds of Molecular Dynamics Simulation before Lunch,” in SC '21: The International Conference for High Performance Computing, Networking, Storage and Analysis. New York, NY, United States: Association for Computing Machinery. 10.1145/3458817.3487397

[B158] Shen L. Yang W. (2018). Molecular Dynamics Simulations with Quantum Mechanics/Molecular Mechanics and Adaptive Neural Networks. J. Chem. Theory Comput. 14, 1442–1455. 10.1021/acs.jctc.7b01195 29438614 PMC6233882

[B159] Shirts M. R. Chodera J. D. (2008). Statistically Optimal Analysis of Samples from Multiple Equilibrium States. J. Chem. Phys. 129, 124105. 10.1063/1.2978177 19045004 PMC2671659

[B160] Singh R. Wiseman B. Deemagarn T. Jha V. Switala J. Loewen P. C. (2008). Comparative Study of Catalase-Peroxidases (KatGs). Archives Biochem. Biophysics 471, 207–214. 10.1016/j.abb.2007.12.008 18178143

[B207] Singhal N. Snow C. D. Pande V. S. (2004). Using Path Sampling to Build Better Markovian State Models: Predicting the Folding Rate and Mechanism of a Tryptophan Zipper Beta Hairpin. J. Chem. Phys. 121, 415–425. 10.1063/1.1738647 15260562

[B161] Sinko W. Miao Y. de Oliveira C. A. F. McCammon J. A. (2013). Population Based Reweighting of Scaled Molecular Dynamics. J. Phys. Chem. B 117, 12759–12768. 10.1021/jp401587e 23721224 PMC3808002

[B162] Sittel F. Stock G. (2018). Perspective: Identification of Collective Variables and Metastable States of Protein Dynamics. J. Chem. Phys. 149, 150901. 10.1063/1.5049637 30342445

[B163] Spiriti J. Wong C. F. (2021). Qualitative Prediction of Ligand Dissociation Kinetics from Focal Adhesion Kinase Using Steered Molecular Dynamics. Life 11, 74–19. 10.3390/life11020074 33498237 PMC7909260

[B164] Stelzl L. S. Kells A. Rosta E. Hummer G. (2017). Dynamic Histogram Analysis to Determine Free Energies and Rates from Biased Simulations. J. Chem. Theory Comput. 13, 6328–6342. 10.1021/acs.jctc.7b00373 29059525

[B165] Stocker S. Csányi G. Reuter K. Margraf J. T. (2020). Machine Learning in Chemical Reaction Space. Nat. Commun. 11, 1–11. 10.1038/s41467-020-19267-x 33127879 PMC7603480

[B166] Suárez E. Wiewiora R. P. Wehmeyer C. Noé F. Chodera J. D. Zuckerman D. M. (2021). What Markov State Models Can and Cannot Do: Correlation versus Path-Based Observables in Protein-Folding Models. J. Chem. Theory Comput. 17, 3119–3133. 10.1021/acs.jctc.0c01154 33904312 PMC8127341

[B167] Svensson F. Engen K. Lundbäck T. Larhed M. Sköld C. (2015). Virtual Screening for Transition State Analogue Inhibitors of IRAP Based on Quantum Mechanically Derived Reaction Coordinates. J. Chem. Inf. Model. 55, 1984–1993. 10.1021/acs.jcim.5b00359 26252078

[B168] Tang Z. Chang C.-e. A. (2018). Binding Thermodynamics and Kinetics Calculations Using Chemical Host and Guest: A Comprehensive Picture of Molecular Recognition. J. Chem. Theory Comput. 14, 303–318. 10.1021/acs.jctc.7b00899 29149564 PMC5920803

[B169] Teo I. Mayne C. G. Schulten K. Lelièvre T. (2016). Adaptive Multilevel Splitting Method for Molecular Dynamics Calculation of Benzamidine-Trypsin Dissociation Time. J. Chem. Theory Comput. 12, 2983–2989. 10.1021/acs.jctc.6b00277 27159059 PMC5724379

[B170] Tiwary P. Mondal J. Berne B. J. (2017). How and when Does an Anticancer Drug Leave its Binding Site? Sci. Adv. 3, e1700014. 10.1126/sciadv.1700014 28580424 PMC5451192

[B171] Tiwary P. Parrinello M. (2015). A Time-independent Free Energy Estimator for Metadynamics. J. Phys. Chem. B 119, 736–742. 10.1021/jp504920s 25046020

[B172] Tiwary P. Parrinello M. (2013). From Metadynamics to Dynamics. Phys. Rev. Lett. 111, 230602. 10.1103/PhysRevLett.111.230602 24476246

[B173] Truhlar D. G. Garrett B. C. Klippenstein S. J. (1996). Current Status of Transition-State Theory. J. Phys. Chem. 100, 12771–12800. 10.1021/jp953748q

[B174] Unke O. T. Chmiela S. Sauceda H. E. Gastegger M. Poltavsky I. Schütt K. T. (2021). Machine Learning Force Fields. Chem. Rev. 121, 10142–10186. 10.1021/acs.chemrev.0c01111 33705118 PMC8391964

[B175] Van Der Velden W. J. C. Heitman L. H. Rosenkilde M. M. (2020). Perspective: Implications of Ligand-Receptor Binding Kinetics for Therapeutic Targeting of G Protein-Coupled Receptors. ACS Pharmacol. Transl. Sci. 3, 179–189. 10.1021/acsptsci.0c00012 32296761 PMC7155193

[B176] Van Erp T. S. Moroni D. Bolhuis P. G. (2003). A Novel Path Sampling Method for the Calculation of Rate Constants. J. Chem. Phys. 118, 7762–7774. 10.1063/1.1562614

[B177] Vanommeslaeghe K. Hatcher E. Acharya C. Kundu S. Zhong S. Shim J. (2011). CHARMM General Force Field: A Force Field for Drug-like Molecules Compatible with the CHARMM All-Atom Additive Biological Force Fields. J. Comput. Chem. 31, 671–690. 10.1002/jcc.21367.CHARMM PMC2888302 19575467

[B178] Vauquelin G. Bostoen S. Vanderheyden P. Seeman P. (2012). Clozapine, Atypical Antipsychotics, and the Benefits of Fast-Off D2 Dopamine Receptor Antagonism. Schmiedeb. Arch. Pharmacol. 385, 337–372. 10.1007/s00210-012-0734-2 22331262

[B179] Vitalini F. Mey A. S. J. S. Noé F. Keller B. G. (2015). Dynamic Properties of Force Fields. J. Chem. Phys. 142, 084101. 10.1063/1.4909549 25725706

[B180] Votapka L. W. Jagger B. R. Heyneman A. L. Amaro R. E. (2017). SEEKR: Simulation Enabled Estimation of Kinetic Rates, A Computational Tool to Estimate Molecular Kinetics and its Application to Trypsin-Benzamidine Binding. J. Phys. Chem. B 121, 3597–3606. SEEKR. 10.1021/acs.jpcb.6b09388 28191969 PMC5562489

[B181] Voter A. F. Doll J. D. (1985). Dynamical Corrections to Transition State Theory for Multistate Systems: Surface -Self-Diffusion in the Rare-Event Regime. J. Chem. Phys. 82, 80–92. 10.1063/1.448739

[B182] Voter A. F. (1997). Hyperdynamics: Accelerated Molecular Dynamics of Infrequent Events. Phys. Rev. Lett. 78, 3908–3911. 10.1103/PhysRevLett.78.3908

[B183] Wan H. Voelz V. A. (2020). Adaptive Markov State Model Estimation Using Short Reseeding Trajectories. J. Chem. Phys. 152, 024103. 10.1063/1.5142457 31941308 PMC7047717

[B184] Wang F. Landau D. P. (2001). Efficient, Multiple-Range Random Walk Algorithm to Calculate the Density of States. Phys. Rev. Lett. 86, 2050–2053. 10.1103/PhysRevLett.86.2050 11289852

[B185] Wang J. Miao Y. (2020). Peptide Gaussian Accelerated Molecular Dynamics (Pep-GaMD): Enhanced Sampling and Free Energy and Kinetics Calculations of Peptide Binding. J. Chem. Phys. 153, 154109. 10.1063/5.0021399 33092378 PMC7575327

[B186] Wang J. Wang W. Kollman P. A. Case D. A. (2006). Automatic Atom Type and Bond Type Perception in Molecular Mechanical Calculations. J. Mol. Graph. Model. 25, 247–260. 10.1016/j.jmgm.2005.12.005 16458552

[B187] Wang J. Wolf R. M. Caldwell J. W. Kollman P. A. Case D. A. (2004). Development and Testing of a General Amber Force Field. J. Comput. Chem. 25, 1157–1174. 10.1002/jcc.20035 15116359

[B188] Wang L. Wu Y. Deng Y. Kim B. Pierce L. Krilov G. (2015). Accurate and Reliable Prediction of Relative Ligand Binding Potency in Prospective Drug Discovery by Way of a Modern Free-Energy Calculation Protocol and Force Field. J. Am. Chem. Soc. 137, 2695–2703. 10.1021/ja512751q 25625324

[B189] Wang Y. Martins J. M. Lindorff-Larsen K. (2017). Biomolecular Conformational Changes and Ligand Binding: from Kinetics to Thermodynamics. Chem. Sci. 8, 6466–6473. 10.1039/c7sc01627a 29619200 PMC5859887

[B190] Wang Y. Valsson O. Tiwary P. Parrinello M. Lindorff-Larsen K. (2018). Frequency Adaptive Metadynamics for the Calculation of Rare-Event Kinetics. J. Chem. Phys. 149, 072309. 10.1063/1.5024679 30134721

[B191] Wang Z. X. Zhang W. Wu C. Lei H. Cieplak P. Duan Y. (2006). Strike a Balance: Optimization of Backbone Torsion Parameters of AMBER Polarizable Force Field for Simulations of Proteins and Peptides. J. Comput. Chem. 27, 781–790. 10.1002/jcc.20386 16526038 PMC3926949

[B192] Wolf S. Amaral M. Lowinski M. Vallée F. Musil D. Güldenhaupt J. (2019). Estimation of Protein-Ligand Unbinding Kinetics Using Non-equilibrium Targeted Molecular Dynamics Simulations. J. Chem. Inf. Model. 59, 5135–5147. 10.1021/acs.jcim.9b00592 31697501

[B193] Wolf S. Lickert B. Bray S. Stock G. (2020). Multisecond Ligand Dissociation Dynamics from Atomistic Simulations. Nat. Commun. 11, 1–8. 10.1038/s41467-020-16655-1 32522984 PMC7286908

[B194] Wolf S. Stock G. (2018). Targeted Molecular Dynamics Calculations of Free Energy Profiles Using a Nonequilibrium Friction Correction. J. Chem. Theory Comput. 14, 6175–6182. 10.1021/acs.jctc.8b00835 30407810

[B195] Woods C. J. Essex J. W. King M. A. (2003). Enhanced Configurational Sampling in Binding Free-Energy Calculations. J. Phys. Chem. B 107, 13711–13718. 10.1021/jp036162+

[B196] Woods C. J. Manby F. R. Mulholland A. J. (2008). An Efficient Method for the Calculation of Quantum Mechanics/molecular Mechanics Free Energies. J. Chem. Phys. 128, 014109. 10.1063/1.2805379 18190187

[B197] Wu H. Paul F. Wehmeyer C. Noé F. (2016). Multiensemble Markov Models of Molecular Thermodynamics and Kinetics. Proc. Natl. Acad. Sci. U.S.A. 113, E3221–E3230. 10.1073/pnas.1525092113 27226302 PMC4988570

[B198] Xue Y. Yuwen T. Zhu F. Skrynnikov N. R. (2014). Role of Electrostatic Interactions in Binding of Peptides and Intrinsically Disordered Proteins to Their Folded Targets. 1. NMR and MD Characterization of the Complex between the C-Crk N-SH3 Domain and the Peptide Sos. Biochemistry 53, 6473–6495. 10.1021/bi500904f 25207671

[B199] Yang M. Bonati L. Polino D. Parrinello M. (2022). Using Metadynamics to Build Neural Network Potentials for Reactive Events: the Case of Urea Decomposition in Water. Catal. Today 387, 143–149. 10.1016/j.cattod.2021.03.018

[B200] Yue S. Muniz M. C. Calegari Andrade M. F. Zhang L. Car R. Panagiotopoulos A. Z. (2021). When Do Short-Range Atomistic Machine-Learning Models Fall Short? J. Chem. Phys. 154, 034111. 10.1063/5.0031215 33499637

[B201] Yue Z. Wang Z. Voth G. A. (2022). Ion Permeation, Selectivity, and Electronic Polarization in Fluoride Channels. Biophysical J. 121, 1336–1347. 10.1016/j.bpj.2022.02.019 PMC9034187 35151630

[B202] Zhang B. W. Jasnow D. Zuckerman D. M. (2010). The “Weighted Ensemble” Path Sampling Method Is Statistically Exact for a Broad Class of Stochastic Processes and Binning Procedures. J. Chem. Phys. 132, 054107. 10.1063/1.3306345 20136305 PMC2830257

[B203] Zhao L. Ciallella H. L. Aleksunes L. M. Zhu H. (2020). Advancing Computer-Aided Drug Discovery (CADD) by Big Data and Data-Driven Machine Learning Modeling. Drug Discov. Today 25, 1624–1638. 10.1016/j.drudis.2020.07.005 32663517 PMC7572559

[B204] Zuckerman D. M. Chong L. T. (2017). Weighted Ensemble Simulation: Review of Methodology, Applications, and Software. Annu. Rev. Biophys. 46, 43–57. Weighted. 10.1146/annurev-biophys-070816-033834 28301772 PMC5896317

