# Supplementary material for: Enhanced-Sampling Simulations for the Estimation of Ligand Binding Kinetics: Current Status and Perspective
Source: Front Mol Biosci. 2022 Jun 8;9:899805. doi: 10.3389/fmolb.2022.899805 (PMC9216551; doi:10.3389/fmolb.2022.899805)
Supplement: Supplementary file 1 [file DataSheet1.PDF]

SUPPLEMENTARY INFORMATION

SI 1. Details on Metadynamics protocols

In the context of infrequent Metadynamics the two-sample Kolmogorov-Smirnov (KS) test (Massey, 1951; Miller, 1956) can be used to assess the computed k_{off} values. It exploits the fact that the times of escape from long-lived metastable states obey Poisson statistics. In practice, the empirical distribution of residence times is compared to a theoretical rare event distribution, and their agreement is assessed by computing the so-called p-value. (Salvalaglio et al., 2014) If the latter is greater than the significance threshold (usually set at 0.15), the empirical distribution is deemed indistinguishable from the theoretical one, proving the validity of the adopted Metadynamics protocol.

SI 2. Gaussian Mixture-Based Enhanced Sampling

Successful characterization of residence times from infrequent/FA MetaD simulations of ligand unbinding is heavily contingent on the avoidance of bias deposition on the transition state, and faithfully fulfilling this requirement is inherently nontrivial. Aside from the deficiencies of modern force-fields, it may be the case that unintentional tainting of the transition state is a factor that affects the accuracy of MetaD-derived k_{off} values. The Gaussian mixture-based enhanced sampling (GAMBES) method recently proposed by Debnath and Parrinello (Debnath and Parrinello, 2020) provides an elegant means of bypassing these difficulties by constructing the external bias from probability distributions of configurations obtained from short MD simulations originating from the metastable states, such that the bias decays to zero on the fringes of the metastable states. Specifically, the combined probability densities associated with the metastable states are approximated by a Gaussian mixture model, and the exponential tails of the Gaussians are trimmed according to a pre-defined cutoff, thus ensuring that no bias is applied outside of the metastable states. First passage times between metastable states can then be rescaled to obtain unbiased residence times. The method has been applied to predict residence times for a set of 12 Octa-acid host-guest systems belonging to the SAMPL5 challenge, and while experimental values for these systems have not yet been determined, the trends observed in the calculated values for these systems correlate well with previously established trends in binding affinities. (Debnath and Parrinello, 2021)

SI 3. Scaled MD

SI 3.1 Basic Principles

The general protocol consists of simple application of a scaling factor λ to the potential energy of the system, with λ taking a value between 0 and 1. (Mark et al., 1991; Tsujishita et al., 1993) This translates into a flattening of the barriers between minima in the potential energy surface, thus enabling faster transitions between minima and accelerating the exploration of the full PES. Lower values of λ correspond to a stronger degree of scaling, whereby the barriers in the potential energy surface are flattened.

SI 3.2 Applications

The method was recently applied to compute and rank relative residence times for five inhibitors of the hDAAO flavoprotein – an enzyme that is involved in the breakdown of D-serine that is observed in some psychiatric disorders. (Bernetti et al., 2018) The sMD simulations made use of the AMBER99SB-ILDN forcefield (Hornak et al., 2006) and a scaling factor λ= 0.45. A total of 90 trajectories were simulated for the five inhibitors, making a total of 3.4 μs of simulation time. Observed ligand exit times from the trajectories were taken as the calculated residence times t_{calc}, which were normalized with respect to t_{calc} of the ligand with the slowest experimental residence time t_{exp}. The ranking of the normalized residence times was compared to the ranking of their normalized experimental residence times – which were subjected to exponential scaling with λ= 0.45 to facilitate comparison to the calculated values. The authors were able to correctly rank four of the five ligands, with a systematic 2 to 4-fold overestimation of the residence time, with one outlier which had a more significantly overpredicted residence time (by around 13-fold). The accuracy of these results appears to be a significant improvement on those that have been yielded by CV-based methods discussed previously, though attaining agreement between calculated and experimental residence times required normalization of both quantities to make the comparison valid. Additionally, the flattening of the PES in the trajectories make it likely that the unbinding routes taken in the simulation are unphysical, as they occur at a relatively high effective temperature.

A similar sMD protocol (Schuetz et al., 2019) using the same scaling factor and force field was applied to the study the binding kinetics of seven inhibitors of Hsp90, with more efforts

directed towards post-processing of the trajectories to reduce noise introduced by the high effective temperature, and subsequent mapping of the unbinding pathways onto a low dimensional space. In addition to the relative ranking of normalized t_{calc} that were made in the previous study of hDAAO inhibitors, direct comparisons were made between t_{calc} and t_{exp} were made. They were able to correctly reproduce the ranking of six of the seven compounds with a Pearson r^2 coefficient of 0.89, however the direct comparison of t_{calc} and t_{exp} revealed that t_{calc} was systematically underestimated by seven to nine orders of magnitude.

SI 3.3. Selectively scaled sMD with Kramers'-based rate extrapolation

Finally, another approach based on sMD was proposed to predict the initial unbinding time (Deb and Frank, 2019), which the author defined as the first step in the unbinding process that can be modeled as a first-order transition. In this case, only the Lennard-Jones interactions between ligand and water are scaled by a parameter $\lambda > 1$ in order to favor unbinding. The authors ran 100 independent simulations using 4 different values of λ and fitted a simplified Kramers rate theory model to obtain the initial unbinding time (Frank and Andricioaei, 2016). Because these time predictions are not associated to the entire dissociation process but only the first step, the predictions for three different inhibitors of the CDK2 kinase underestimated the residence times by five orders of magnitude.

Overall, evidence of the applicability of sMD to yield quantitative residence times is scant, and much like traditional TMD it appears better suited to relative ranking of lead compounds in early high-throughput screening at present.

SI 4. Targeted MD

SI 4.1 Basic principles

Targeted MD (TMD) makes use of an external steering force, which is applied to a subset of atoms to pull them along a defined pathway or reaction coordinate x. (Schlitter et al., 1994) TMD introduces a periodically updated holonomic constraint force f_c into the simulation, which slowly steers the pull group along x from an initial state x_A to a final state x_B at a constant velocity v_c. For a protein-ligand system, the steering coordinate x is essentially the radial component of a spherical polar coordinate system, which would correspond to the distance

between the center-of-mass of the binding pocket and that of the ligand. Thus, the ligand is able to explore configurational space perpendicular to x, and can freely perform rotations and conformational changes. Ideally, v_c is much slower than the other degrees of freedom of the ligand, such that v_c is unaffected by friction forces and acceleration due to free energy gradients. Running large ensembles of TMD simulations ensures that the effect of stochastic forces on v_c are effectively eliminated. Integrating f_c along x for all trajectories in the ensemble yields the ensemble-averaged nonequilibrium work $\langle W \rangle$ performed on the system. This is then used to obtain the free energy profile $\Delta G(x)$ directly, as shown by Wolf and Stock. (Wolf and Stock, 2018)

SI 4.2 Applications

A TMD protocol using the Amber99SB (Hornak et al., 2006) force field was applied to investigate the correlation between the $\langle W \rangle$ and the experimental k_{off} for a set of twenty-six potential inhibitors of the chaperone heat shock protein Hsp90; some of which bound to helix domains, while others bound to loop domains. Fitting the full set of calculated $\langle W \rangle$ values for all of the ligands against their experimental k_{off} yielded a low Pearson's correlation coefficient $r^2 = 0.45$. However, when the nine helix binders were considered alone, the correlation between $\langle W \rangle$ and experimental k_{off} for these compounds was relatively strong, with an r^2 value of 0.80. These results indicate that TMD could be useful for initial qualitative ranking of site-specific lead compounds in high-throughput screenings, especially since each prediction required only 10 ns of aggregate TMD simulation time.

SI 5. Qualitative applications of τRAMD

In the first application of the τRAMD method, a set of 70 drug-like compounds binding to Hsp90α were investigated and ranked according to their computed relative residence times. (Kokh et al., 2018) The simulations made use of the AMBER14 force field (Maier et al., 2015) for the protein in conjunction with GAFF(Wang et al., 2004) for the ligands, and force of magnitude 14 kcal $mol^{-1}Å^{-1}$ was applied to the ligand at each 100 fs checkpoint. Very good correlation ($r^2 = 0.86$) between t_{comp} and experimentally measured residence times t_{exp} was achieved for 78% of the compounds, while the residence times for the other 22% of the compounds were underestimated. A follow up study included 25 additional Hsp90α inhibitors, and implemented machine learning protocols on the combined datasets to uncover molecular

determinants of longer residence times, and to predict corrected t_{comp} values for the compounds with underestimated residence times with regression models trained on t_{comp} and experimental (t_{exp}) values for the other compounds. (Kokh et al., 2019) Since 2020, the τRAMD protocol has been fully implemented in the GROMACS MD engine (Abraham et al., 2015) as an open-source solution for the calculation of relative ligand residence times for other systems. (Kokh et al., 2020)

SI 6 Methodological details of MSMs discussed in Section 3

The work of Plattner and Noé (Plattner and Noé, 2015) on the trypsin-benzamidine system in 2015 provides an archetypal example of how MSMs are applied to calculate k_{off} values for ligand-enzyme complexes. The system was modeled using the AMBER99SB(Hornak et al., 2006) force field for the trypsin enzyme, and GAFF(Wang et al., 2004) for the benzamidine ligand, for an aggregate simulation time of 149.1 μs. This was split amongst 491 trajectories of 100 ns duration, 4 of 1 μs duration and 48 of 2 μs duration, all of which were unbiased. A time-lagged independent component analysis (TICA) was performed to reduce the dimensionality of the trajectories by identifying five maximal-variance projection vectors that correspond to the most slowly evolving components of the proteins conformational space. This was followed by uniform distance clustering, to yield a set of 273 microstates for which a transition matrix was computed, using a lag time *t* of 30 ns. Coarse-graining of microstates into metastable macrostates was achieved through Perron-cluster cluster analysis (PCCA++) (Röblitz and Weber, 2013).The mean number of *t* intervals between the all of the macrostates visited from the bound state to the unbound state can then be used to calculate an overall mean first passage time (MFPT) from the bound to the unbound state, and k_{off} as its inverse.

A similar protocol was employed in the investigation of the binding mechanism of T4 lysozyme L99A in complex with benzene. (Mondal et al., 2018) Six extended trajectories in the ms regime and 300 shorter trajectories of unbiased dynamics using the CHARMM36 (Best et al., 2012) force field were collected, yielding 59 μs of gross simulation time from which an MSM with a 10 ns time lag was constructed. After discretization (using the k-means clustering algorithm) of the 12 TICA components of the system, a total of 100 microstates were unearthed. Rather than PCCA++, a hidden Markov model (HMM) (Noé et al., 2013)was employed to yield a macrostate MSM with four metastable states from the 100-state MSM. MFPT values

were extracted from the coarse-grained model, giving a $k_{\text{off}} = 310 \pm 130$ s^{-1}. This k_{off} value was marginally closer to the experimental k_{off} (950 ± 20 s^{-1})(Feher et al., 1996) than the value calculated from their infrequent MetaD simulations (270 ±100 s^{-1})(Mondal et al., 2018), however the larger error range and relatively large amount of simulation time required possibly offsets this benefit.

SI 7. A highly scalable QM/MM code

A highly scalable QM/MM code has been developed by our group at Juelich in collaboration with a large European consortium. This is the so-called MiMiC interface (Bolnykh et al., 2019; Olsen et al., 2019), which allows subnanosecond QM/MM MD simulations of biologically relevant systems. (Chiariello et al., 2020) MiMiC has been designed as a general multiscale simulation framework that enables the combination of multiple resolutions and methods for different parts of a system, while retaining high computational efficiency (Bolnykh et al., 2020a). Improving the scalability of MiMiC could allow for conformational sampling and optimization at the QM/MM level exploiting exascale machines (Rovira, 2013; Bolnykh et al., 2020b).

SI 8. Hybrid machine learning / molecular mechanics potentials

In machine learning / molecular mechanics ML/MM potentials, the energy is decomposed as

$$U = U_{ML} + U_{MM} + U_{ML/MM}$$

where U_{ML}, U_{MM} and $U_{ML/MM}$ are the potential energy resulting from the interactions between atoms within the ML region, within the MM region, and between atoms belonging to different regions respectively. The main challenge for ML/MM simulations is the calculation of $U_{ML/MM}$. A simple solution for computing $U_{ML/MM}$ is inspired by the mechanical embedding approach commonly used in QM/MM simulations. Briefly, the atoms in the ML region are assigned point charges and Lennard-Jones parameters, and $U_{ML/MM}$ is simply computed using the force field, while a neural network (NN) is used exclusively to predict U_{ML} (Lahey and Rowley, 2020; Galvelis et al., 2022) The partial charges can be fixed throughout the simulation, or they can be predicted by the NN itself (Xu et al., 2021) This approach was adopted to

improve the accuracy of relative binding free energy predictions using alchemical methods (Rufa et al., 2020) It has also been applied to modelling metalloproteins (Xu et al., 2021) refining protein-ligand structures from electron density data (Vant et al., 2020) An important advantage of this strategy is that it is in principle compatible with the many existing NN architectures designed for full ML-potential simulations. Moreover, long-ranged interactions are automatically included in the potential at the MM level. On the other hand, polarization and charge transfer effects between the MM and QM regions are ignored. In more recent years, other NN potentials capable of incorporating the effects of the MM environment have been developed. In most of these methods, the NN computes only a correction to a low-level (typically semi-empirical) Hamiltonian, which besides modelling much of the physics at a reasonable cost, comes with established routes to incorporate long-ranged interactions. The main difference between these methods consists in how the MM environment is represented. In one approach, all the MM atoms within a cutoff distance explicitly enter the NN inputs, and they differ from the ML atoms only because they are assigned a specific MM type. (Böselt et al., 2021; Zeng et al., 2021) Other NN architectures model the MM environment implicitly, for example by representing it either with Mulliken charges computed with the low-level potential(Wu et al., 2017), as an external electrostatic potential generated by the MM atoms and defined on a grid (Shen and Yang, 2018), or as an external electric field acting on NN-predicted dipoles.(Gastegger et al., 2021) Another approach based on the representation of the MM environment as both an external potential and electric field was successfully employed to reproduce the free energy profile of an enzymatic reaction at the B3LYP/MM level of theory. (Pan et al., 2017)

References

Abraham, M. J., Murtola, T., Schulz, R., Páll, S., Smith, J. C., Hess, B., et al. (2015). Gromacs: High performance molecular simulations through multi-level parallelism from laptops to supercomputers. *SoftwareX* 1–2, 19–25. doi:10.1016/j.softx.2015.06.001.

Bernetti, M., Rosini, E., Mollica, L., Masetti, M., Pollegioni, L., Recanatini, M., et al. (2018). Binding Residence Time through Scaled Molecular Dynamics: A Prospective Application to hDAAO Inhibitors. *J. Chem. Inf. Model.* 58, 2255–2265. doi:10.1021/acs.jcim.8b00518.

Best, R. B., Zhu, X., Shim, J., Lopes, P. E. M., Mittal, J., Feig, M., et al. (2012). Optimization of the Additive CHARMM All-Atom Protein Force Field Targeting Improved Sampling of the Backbone ϕ, ψ and Side-Chain $\chi 1$ and $\chi 2$ Dihedral Angles. *J. Chem. Theory Comput.* 8, 3257–3273. doi:10.1021/ct300400x.

Bolnykh, V., Olsen, J. M. H., Meloni, S., Bircher, M. P., Ippoliti, E., Carloni, P., et al. (2019). Extreme Scalability of DFT-Based QM/MM MD Simulations Using MiMiC. *J. Chem. Theory Comput.* 15, 5601–5613. doi:10.1021/acs.jctc.9b00424.

Bolnykh, V., Olsen, J. M. H., Meloni, S., Bircher, M. P., Ippoliti, E., Carloni, P., et al. (2020a). MiMiC: Multiscale Modeling in Computational Chemistry. *Front. Mol. Biosci.* 7, 1–4. doi:10.3389/fmolb.2020.00045.

Bolnykh, V., Rothlisberger, U., and Carloni, P. (2020b). Biomolecular Simulation: A Perspective from High Performance Computing. *Isr. J. Chem.* 60, 694–704.

Böselt, L., Thürlemann, M., and Riniker, S. (2021). Machine Learning in QM/MM Molecular Dynamics Simulations of Condensed-Phase Systems. *J. Chem. Theory Comput.* 17, 2641–2658. doi:10.1021/acs.jctc.0c01112.

Chiariello, M. G., Bolnykh, V., Ippoliti, E., Meloni, S., Olsen, J. M. H., Beck, T., et al. (2020). Molecular Basis of CLC Antiporter Inhibition by Fluoride. *J. Am. Chem. Soc.* 142, 7254–7258. doi:10.1021/jacs.9b13588.

Deb, I., and Frank, A. T. (2019). Accelerating Rare Dissociative Processes in Biomolecules Using Selectively Scaled MD Simulations. *J. Chem. Theory Comput.* 15, 5817–5828. doi:10.1021/acs.jctc.9b00262.

Feher, V. A., Baldwin, E. P., and Dahlquist, F. W. (1996). Access of ligands to cavities within the core of a protein is rapid. 3, 516–521.

Frank, A. T., and Andricioaei, I. (2016). Reaction Coordinate-Free Approach to Recovering Kinetics from Potential-Scaled Simulations: Application of Kramers' Rate Theory. *J.*

Phys. Chem. B 120, 8600–8605. doi:10.1021/acs.jpcb.6b02654.

Galvelis, R., Varela-Rial, A., Doerr, S., Fino, R., Eastman, P., Markland, T. E., et al. (2022). NNP/MM: Fast molecular dynamics simulations with machine learning potentials and molecular mechanics. 1–21. Available at: http://arxiv.org/abs/2201.08110.

Gastegger, M., Schütt, K. T., and Müller, K. R. (2021). Machine learning of solvent effects on molecular spectra and reactions. *Chem. Sci.* 12, 11473–11483. doi:10.1039/d1sc02742e.

Hornak, V., Abel, R., Okur, A., Strockbine, B., Roitberg, A., and Simmerling, C. (2006). Comparison of multiple Amber force fields and development of improved protein backbone parameters. *Proteins* 65, 712–725. doi:10.1002/prot.21123.

Kokh, D. B., Amaral, M., Bomke, J., Grädler, U., Musil, D., Buchstaller, H. P., et al. (2018). Estimation of Drug-Target Residence Times by τ-Random Acceleration Molecular Dynamics Simulations. *J. Chem. Theory Comput.* 14, 3859–3869. doi:10.1021/acs.jctc.8b00230.

Kokh, D. B., Doser, B., Richter, S., Ormersbach, F., Cheng, X., and Wade, R. C. (2020). A workflow for exploring ligand dissociation from a macromolecule: Efficient random acceleration molecular dynamics simulation and interaction fingerprint analysis of ligand trajectories. *J. Chem. Phys.* 153. doi:10.1063/5.0019088.

Kokh, D. B., Kaufmann, T., Kister, B., and Wade, R. C. (2019). Machine learning analysis of τRAMD trajectories to decipher molecular determinants of drug-target residence times. *Front. Mol. Biosci.* 6, 1–17. doi:10.3389/fmolb.2019.00036.

Lahey, S. L. J., and Rowley, C. N. (2020). Simulating protein-ligand binding with neural network potentials. *Chem. Sci.* 11, 2362–2368. doi:10.1039/c9sc06017k.

Maier, J. A., Martinez, C., Kasavajhala, K., Wickstrom, L., Hauser, K. E., and Simmerling, C. (2015). ff14SB: Improving the Accuracy of Protein Side Chain and Backbone Parameters from ff99SB. *J. Chem. Theory Comput.* 11, 3696–3713. doi:10.1021/acs.jctc.5b00255.

Mark, A. E., van Gunsteren, W. F., and Berendsen, H. J. C. (1991). Calculation of relative free energy via indirect pathways. *J. Chem. Phys.* 94, 3808–3816. doi:10.1063/1.459753.

Massey, F. J. (1951). The Kolmogorov-Smirnov Test for Goodness of Fit. *J. Am. Stat. Assoc.* 46, 68–78. doi:10.2307/2280095.

Miller, L. H. (1956). Table of Percentage Points of Kolmogorov Statistics. *J. Am. Stat. Assoc.* 51, 111–121. doi:10.1080/01621459.1956.10501314.

Mondal, J., Ahalawat, N., Pandit, S., Kay, L. E., and Vallurupalli, P. (2018). Atomic resolution mechanism of ligand binding to a solvent inaccessible cavity in T4 lysozyme. *PLoS Comput. Biol.* 14, 1–20. doi:10.1371/journal.pcbi.1006180.

Noé, F., Wu, H., Prinz, J. H., and Plattner, N. (2013). Projected and hidden Markov models for calculating kinetics and metastable states of complex molecules. *J. Chem. Phys.* 139. doi:10.1063/1.4828816.

Olsen, J. M. H., Bolnykh, V., Meloni, S., Ippoliti, E., Bircher, M. P., Carloni, P., et al. (2019). MiMiC: A Novel Framework for Multiscale Modeling in Computational Chemistry. *J. Chem. Theory Comput.* 15, 3810–3823. doi:10.1021/acs.jctc.9b00093.

Pan, A. C., Xu, H., Palpant, T., and Shaw, D. E. (2017). Quantitative Characterization of the Binding and Unbinding of Millimolar Drug Fragments with Molecular Dynamics Simulations. *J. Chem. Theory Comput.* 13, 3372–3377. doi:10.1021/acs.jctc.7b00172.

Plattner, N., and Noé, F. (2015). Protein conformational plasticity and complex ligand-binding kinetics explored by atomistic simulations and Markov models. *Nat. Commun.* 6. doi:10.1038/ncomms8653.

Röblitz, S., and Weber, M. (2013). Fuzzy spectral clustering by PCCA+: application to Markov state models and data classification. *Adv. Data Anal. Classif.* 7, 147–179. Available at: https://econpapers.repec.org/RePEc:spr:advdac:v:7:y:2013:i:2:p:147-179.

Rovira, C. (2013). The description of electronic processes inside proteins from Car–Parrinello molecular dynamics: chemical transformations. *Wiley Interdiscip. Rev. Comput. Mol. Sci.* 3, 393–407.

Rufa, D. A., Bruce Macdonald, H. E., Fass, J., Wieder, M., Grinaway, P. B., Roitberg, A. E., et al. (2020). Towards chemical accuracy for alchemical free energy calculations with hybrid physics-based machine learning / molecular mechanics potentials. *bioRxiv*. doi:10.1101/2020.07.29.227959.

Salvalaglio, M., Tiwary, P., and Parrinello, M. (2014). Assessing the Reliability of the Dynamics Reconstructed from Metadynamics. *J. Chem. Theory Comput.* 10, 1420–1425. doi:10.1021/ct500040r.

Schlitter, J., Engels, M., and Krüger, P. (1994). Targeted molecular dynamics: A new approach for searching pathways of conformational transitions. *J. Mol. Graph.* 12, 84–89.

Schuetz, D. A., Bernetti, M., Bertazzo, M., Musil, D., Eggenweiler, H. M., Recanatini, M., et al. (2019). Predicting Residence Time and Drug Unbinding Pathway through Scaled Molecular Dynamics. *J. Chem. Inf. Model.* 59, 535–549. doi:10.1021/acs.jcim.8b00614.

Shen, L., and Yang, W. (2018). Molecular Dynamics Simulations with Quantum Mechanics/Molecular Mechanics and Adaptive Neural Networks. *J. Chem. Theory Comput.* 14, 1442–1455. doi:10.1021/acs.jctc.7b01195.

Tsujishita, H., Moriguchi, I., and Hirono, S. (1993). Potential-scaled molecular dynamics and potential annealing: effective conformational search techniques for biomolecules. *J. Phys. Chem.* 97, 4416–4420. doi:10.1021/j100119a028.

Vant, J. W., Lahey, S.-L. J., Jana, K., Shekhar, M., Sarkar, D., Munk, B. H., et al. (2020). Flexible Fitting of Small Molecules into Electron Microscopy Maps Using Molecular Dynamics Simulations with Neural Network Potentials. *J. Chem. Inf. Model.* 60, 2591–2604. doi:10.1021/acs.jcim.9b01167.

Wang, J., Wolf, R. M., Caldwell, J. W., Kollman, P. A., and Case, D. A. (2004). Development and testing of a general amber force field. *J. Comput. Chem.* 25, 1157–1174. doi:10.1002/jcc.20035.

Wolf, S., and Stock, G. (2018). Targeted Molecular Dynamics Calculations of Free Energy Profiles Using a Nonequilibrium Friction Correction. *J. Chem. Theory Comput.* 14, 6175–6182. doi:10.1021/acs.jctc.8b00835.

Wu, J., Shen, L., and Yang, W. (2017). Internal force corrections with machine learning for quantum mechanics/molecular mechanics simulations. *J. Chem. Phys.* 147, 161732. doi:10.1063/1.5006882.

Xu, M., Zhu, T., and Zhang, J. Z. H. (2021). Automatically Constructed Neural Network Potentials for Molecular Dynamics Simulation of Zinc Proteins. *Front. Chem.* 9. doi:10.3389/fchem.2021.692200.

Zeng, J., Giese, T. J., Ekesan, Ş., and York, D. M. (2021). Development of Range-Corrected Deep Learning Potentials for Fast, Accurate Quantum Mechanical/Molecular Mechanical Simulations of Chemical Reactions in Solution. *J. Chem. Theory Comput.* 17, 6993–7009. doi:10.1021/acs.jctc.1c00201.
